# Transcriptomics-Driven Characterization of LUZ100, a T7-like Pseudomonas Phage with Temperate Features

**DOI:** 10.1128/msystems.01189-22

**Published:** 2023-02-16

**Authors:** Leena Putzeys, Jorien Poppeliers, Maarten Boon, Cédric Lood, Marta Vallino, Rob Lavigne

**Affiliations:** a Laboratory of Gene Technology, Department of Biosystems, KU Leuven, Leuven, Belgium; b Institute for Sustainable Plant Protection, CNR, Turin, Italy; Technical University of Denmark

**Keywords:** nanopore sequencing, *Pseudomonas aeruginosa*, T7-like phages, bacteriophages, transcriptomics

## Abstract

*Autographiviridae* is a diverse yet distinct family of bacterial viruses marked by a strictly lytic lifestyle and a generally conserved genome organization. Here, we characterized Pseudomonas aeruginosa phage LUZ100, a distant relative of type phage T7. LUZ100 is a podovirus with a limited host range which likely uses lipopolysaccharide (LPS) as a phage receptor. Interestingly, infection dynamics of LUZ100 indicated moderate adsorption rates and low virulence, hinting at temperate characteristics. This hypothesis was supported by genomic analysis, which showed that LUZ100 shares the conventional T7-like genome organization yet carries key genes associated with a temperate lifestyle. To unravel the peculiar characteristics of LUZ100, ONT-cappable-seq transcriptomics analysis was performed. These data provided a bird’s-eye view of the LUZ100 transcriptome and enabled the discovery of key regulatory elements, antisense RNA, and transcriptional unit structures. The transcriptional map of LUZ100 also allowed us to identify new RNA polymerase (RNAP)-promoter pairs that can form the basis for biotechnological parts and tools for new synthetic transcription regulation circuitry. The ONT-cappable-seq data revealed that the LUZ100 integrase and a MarR-like regulator (proposed to be involved in the lytic/lysogeny decision) are actively cotranscribed in an operon. In addition, the presence of a phage-specific promoter transcribing the phage-encoded RNA polymerase raises questions on the regulation of this polymerase and suggests that it is interwoven with the MarR-based regulation. This transcriptomics-driven characterization of LUZ100 supports recent evidence that T7-like phages should not automatically be assumed to have a strictly lytic life cycle.

**IMPORTANCE** Bacteriophage T7, considered the “model phage” of the *Autographiviridae* family, is marked by a strictly lytic life cycle and conserved genome organization. Recently, novel phages within this clade have emerged which display characteristics associated with a temperate life cycle. Screening for temperate behavior is of utmost importance in fields like phage therapy, where strictly lytic phages are generally required for therapeutic applications. In this study, we applied an omics-driven approach to characterize the T7-like Pseudomonas aeruginosa phage LUZ100. These results led to the identification of actively transcribed lysogeny-associated genes in the phage genome, pointing out that temperate T7-like phages are emerging more frequent than initially thought. In short, the combination of genomics and transcriptomics allowed us to obtain a better understanding of the biology of nonmodel *Autographiviridae* phages, which can be used to optimize the implementation of phages and their regulatory elements in phage therapy and biotechnological applications, respectively.

## INTRODUCTION

As of January 2023, over 700 complete genome sequences of Pseudomonas phages, mostly infecting Pseudomonas aeruginosa, are available through the United States National Center for Biotechnology Information (NCBI). These phages display extensive morphological and genomic diversity and have distinctive replication strategies (1–3). The majority of these phages belong to the *Caudoviricetes*, the class of tailed, double-stranded DNA phages. Among these, the *Autographiviridae* family currently encompasses nine subfamilies and 133 genera (4). The hallmark feature of the members of this family is that they encode a single-subunit RNA polymerase (RNAP), enabling the phage to self-transcribe its own genes. In addition, all *Autographiviridae* phages display considerable synteny across their genomes.

Escherichia virus T7, one of the best studied bacterial viruses and type species of the subfamily *Studiervirinae* (genus *Teseptimavirus*), was instrumental in unravelling the molecular underpinnings of DNA replication and contributed greatly to the development of molecular biology in general (5). Specifically, its unique transcriptional scheme has become an essential tool in the field of microbial synthetic biology (6). The T7 RNA polymerase and its cognate promoter sequence are used to design highly controlled genetic circuits and drive the expression of desired gene products in various hosts.

T7-like phages have long been considered to replicate by a strictly lytic lifestyle. However, their distinctive genome organization was found as a prophage in Pseudomonas putida KT2440, suggesting that certain *Autographiviridae* phages might be prone to a lysogenic lifestyle (7). Indeed, some T7-like phages have been identified that encoded a site-specific integrase in close proximity to the RNA polymerase gene (8–14). It was only recently demonstrated that certain T7-like phages that infect *Pelagibacter* and *Agrobacterium* do lysogenize their host cells (14, 15).

Here, we describe a novel phage targeting P. aeruginosa, LUZ100, a distant relative of the genus *Teseptimavirus* in the *Autographiviridae* family that harbors genes associated with a temperate lifestyle. This phage is, to the best of our knowledge, the first representative of its kind that can infect the pathogen P. aeruginosa. We characterized its morphological and biological properties, performed a genomic analysis, and charted the LUZ100 transcriptional landscape using the recently established ONT-cappable-seq method (16). This nanopore-based RNA sequencing method enables full-length profiling of primary prokaryotic transcripts and provides a global map of key viral regulatory sequences involved in transcriptional reprogramming of the host cell.

## RESULTS AND DISCUSSION

### Biological characteristics of phage LUZ100. (i) Phage morphology and host range.

**FIG 1:**
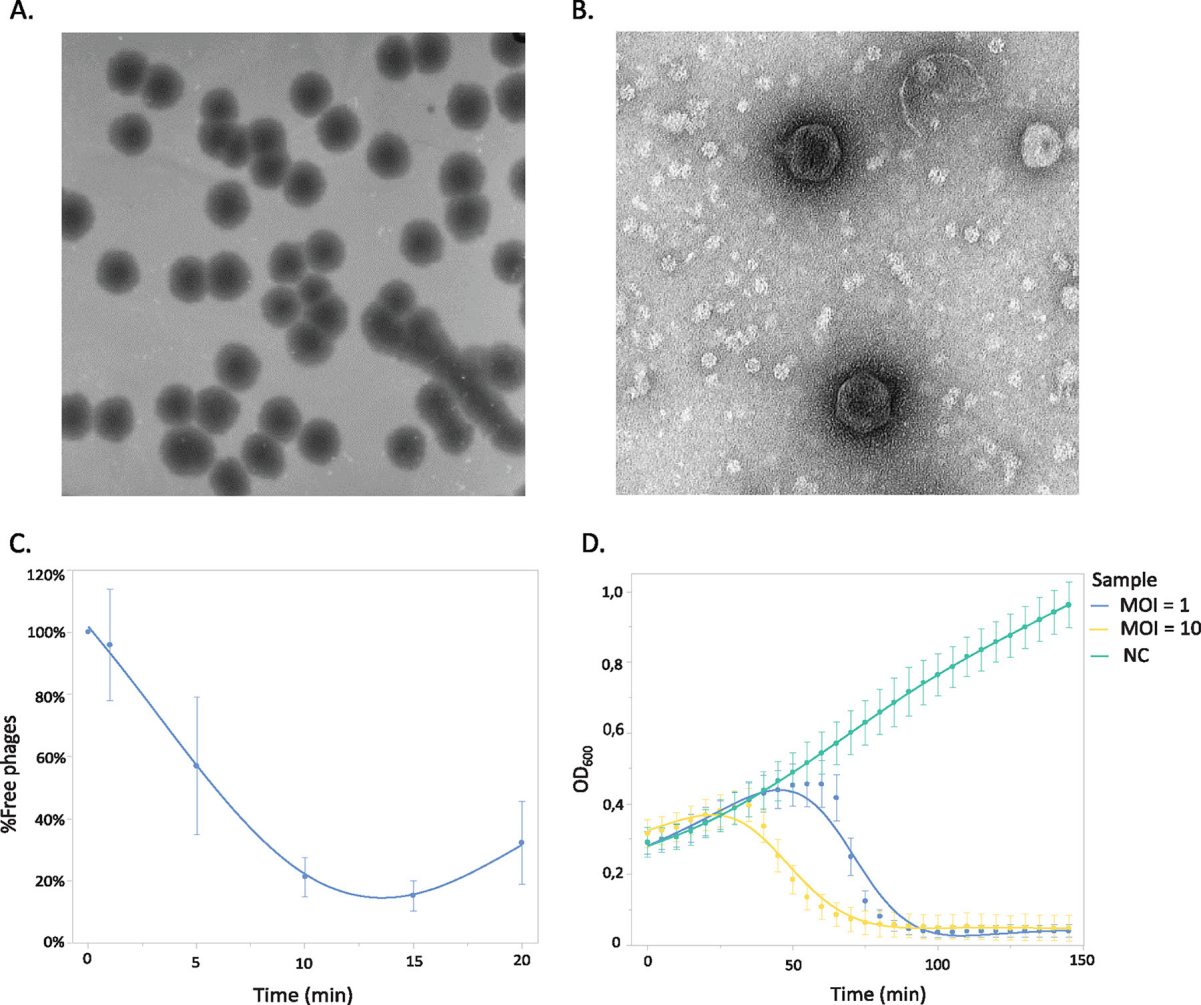
Morphological and microbiological features of phage LUZ100. (A) Plaques of LUZ100 on P. aeruginosa PaLo41, showing large bull’s-eye plaques (B) Transmission electron microscopy image demonstrating the podovirus virion morphology of LUZ100. (C) Adsorption curve of LUZ100 on P. aeruginosa PaLo41, showing that 85% of the phages is adsorbed to the host cell surface after 15 min. (D) Infection curves of LUZ100 infecting P. aeruginosa PaLo41. At an MOI of 10, LUZ100 completes its infection cycle after approximately 30 min, causing lysis of the bacterial cells. NC, negative control.

Phage LUZ100 was isolated from hospital sewage water using clinical P. aeruginosa strain PaLo41 as the host bacterium. After being cultured on a bacterial lawn of PaLo41, LUZ100 produces plaques of 4 to 5 mm, surrounded by an opaque halo zone of approximately 1 to 2 mm (Fig. 1A). Transmission electron microscopy analysis confirms that LUZ100 is a podovirus with a short stubby tail attached to an icosahedral head (Fig. 1B).

The host range and lytic activity of the phage were determined for a diverse collection of 74 P. aeruginosa isolates, including 71 clinical strains and the laboratory strains PAO1k, PA7, and PA14. Phage LUZ100 displays a narrow host spectrum, infecting 23% of the strains in the panel (see Fig. S2 in the supplemental material).

10.1128/msystems.01189-22.9 FIG S2 Host range analysis of LUZ100. The susceptibility to LUZ100 phage infection was tested for 47 clinical isolates (PaLo1 to -47), 24 isolates from the Pirnay collection, and three reference strains (PAO1k, PA7, and PA14). The light green color indicates that the phage lysate induced a bactericidal effect on the bacterial lawn, without generating plaques, while the dark green color shows clearing of the bacterial lawn with the formation of plaques. The pink color corresponds to a lack of infection. Download FIG S2, PDF file, 0.08 MB. Copyright © 2023 Putzeys et al. 2023 Putzeys et al. https://creativecommons.org/licenses/by/4.0/ This content is distributed under the terms of the Creative Commons Attribution 4.0 International license.

### (ii) Phage adsorption rate.

An adsorption assay was performed to assess the efficiency and timing of LUZ100 phage infection. After 15 min, 85% of the phages were adsorbed to the host cell surface (Fig. 1C). The average adsorption constants (*k*) after 1 and 5 min equal 8.00 × 10^−9 ^mL/min and 2.63 × 10^−9 ^mL/min, respectively. In comparison to other members of the T7-like phages, such as P. putida phage phi15 (*k* = 2.51 × 10^−8 ^mL/min after 1 min) and P. fluorescens phage IBB-PF7A (*k* = 5.58 × 10^−10 ^mL/min after 5 min), LUZ100 has a moderate adsorption rate (17, 18).

### (iii) Infection curves.

LUZ100 infection curves of PaLo41 infected at different multiplicities of infection (MOI) were analyzed to determine when bacteria begin to lyse after phage infection (Fig. 1D). At an MOI of 10, the optical density decreases after approximately 30 min, marking the completion of the LUZ100 infection cycle. In addition, the virulence of LUZ100 was assessed by calculating the virulence index and phage score. These metrics equal 0.069 and 0.065, respectively, which is low in contrast to those of the strictly lytic phage T7 (virulence index of 0.84) (19, 20). The reduced LUZ100 lytic activity is logically related to its potential temperate character, discussed below. However, attempts to generate stable lysogens have not been successful so far (Fig. S1).

10.1128/msystems.01189-22.8 FIG S1 Lysogen generation. Purified phage-resistant colonies of individual P. aeruginosa strains PaLo41, PaLo44, PaLo226, PaLo249, PaLo287, and PaLo402 were screened for the presence of LUZ100 by PCR with phage-specific primers. After the reaction, the PCR samples were loaded on an agarose gel to validate the presence of an amplified product. L, 1-kb GeneRuler ladder; +, positive control, LUZ100 DNA (10 ng). Download FIG S1, PDF file, 0.1 MB. Copyright © 2023 Putzeys et al. 2023 Putzeys et al. https://creativecommons.org/licenses/by/4.0/ This content is distributed under the terms of the Creative Commons Attribution 4.0 International license.

### (iv) Identification of phage receptor.

Whole-genome sequencing of four spontaneous phage-resistant PaLo41 mutants was carried out to identify the bacterial receptor to which LUZ100 binds on its host. When compared to the host genome, two of the four mutants showed single nucleotide polymorphisms (SNPs) in the coding sequence of O-antigen ligase (WaaL) (Table S3). WaaL is involved in the lipopolysaccharide (LPS) biosynthesis pathway (21). This hints at LPS as the likely receptor for phage LUZ100. This is consistent with several other members of the T7-like phages, including gh-1 and T7, which recognize LPS as the primary receptor. However, it differs from other known T7-like Pseudomonas phages, including phiKMV and LUZ19, which use type IV pili as primary receptors (22–25).

10.1128/msystems.01189-22.3 TABLE S3 Single nucleotide polymorphisms found in each of the four phage-resistant PaLo41 mutants. Download Table S3, PDF file, 0.1 MB. Copyright © 2023 Putzeys et al. 2023 Putzeys et al. https://creativecommons.org/licenses/by/4.0/ This content is distributed under the terms of the Creative Commons Attribution 4.0 International license.

### Genomic and transcriptomic analysis of LUZ100. (i) General genomic features of LUZ100.

**FIG 2:**
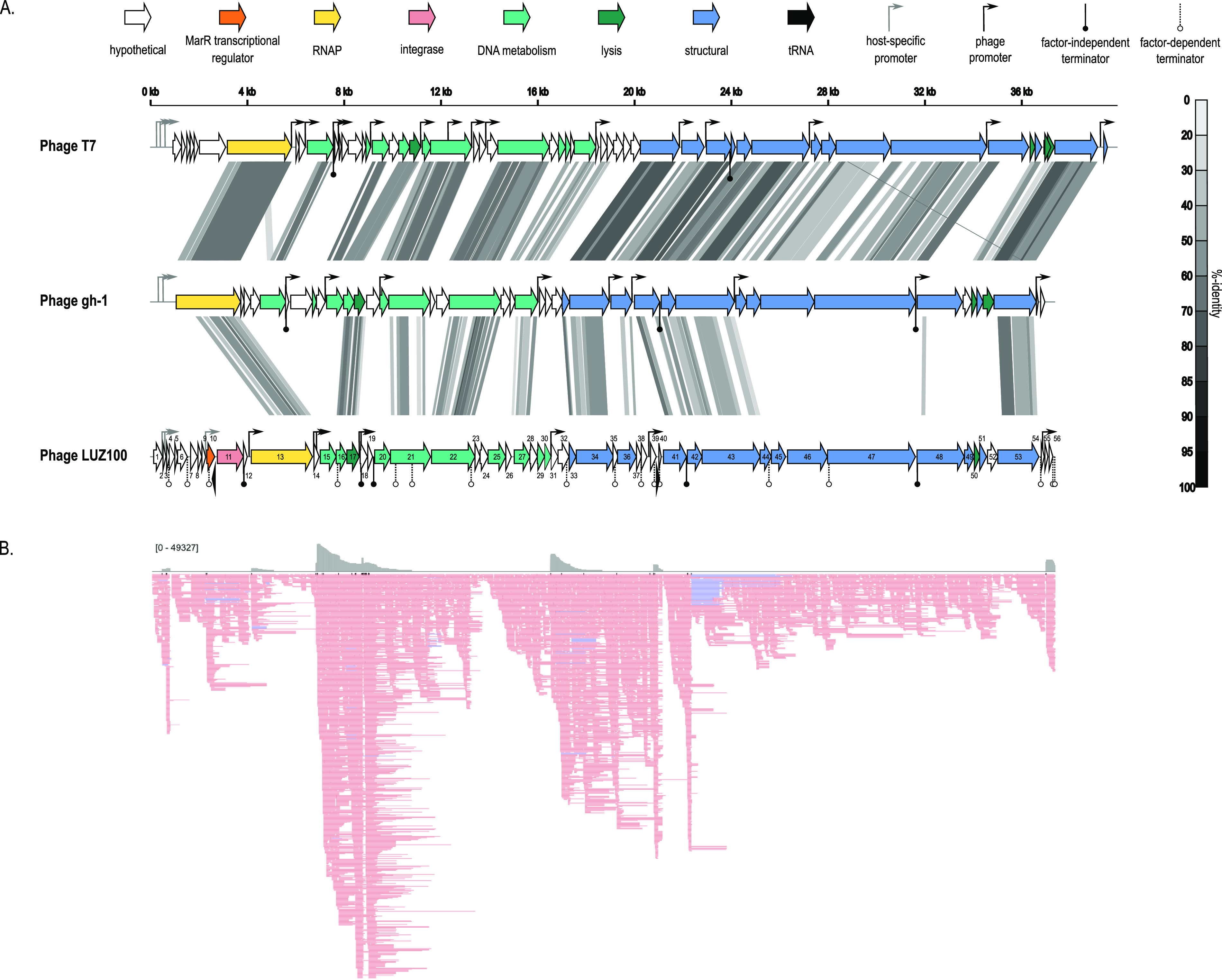
Genomic and transcriptomic overview of Pseudomonas phage LUZ100. (A) Comparison of the genomes of phage T7, gh-1, and the novel Pseudomonas phage LUZ100. Each arrow represents an ORF. The tRNA genes (black), phage RNAP (yellow), and genes involved in DNA metabolism (green), virion structure (blue), lysis (dark green), and integration (pink) are highlighted. Pairwise genome comparisons were generated by tBLASTx in VipTree (59) and show the percent identity of similar regions in grayscale. Promoters are indicated with rightward arrows, where the host-specific promoters (gray) and phage RNAP-specific promoters (black) are marked in different colors. Circles below the genomic map represent terminators, with putative factor-independent terminator sequences indicated in black. (B) ONT-cappable-seq data track of the transcriptomic landscape of phage LUZ100 visualized in IGV (downsampling with a 10-base window size, 50 reads per window). The upper part represents the coverage plot and the lower part visualizes the read alignments. Reads that align to the Watson strand and Crick strand are indicated in pink and blue, respectively.

LUZ100 has a linear double-stranded DNA genome containing 37,343 bp, flanked by 244-bp direct terminal repeats. The genome has an average GC content of 61% and contains 56 open reading frames (ORFs) and two tRNAs (Table S4). The biological functions of 30 translated ORFs could be predicted, leaving 26 ORFs assigned as hypothetical proteins. The predicted features and overall genomic organization of phage LUZ100 reveal striking similarity to members of the *Studiervirinae* in the phage family *Autographiviridae*, including coliphage T7 (NC_001604) and Pseudomonas phage gh-1 (NC_004665.1) (22) (Fig. 2A). Similar to the canonical T7 gene order, the LUZ100 genome can be roughly organized into three functional and temporal modules, representing the genomic regions involved in host takeover (early), DNA metabolism (middle), and virion morphogenesis (late) (26). However, at the nucleotide level, the genomic sequence of LUZ100 is unique, showing only distant sequence similarity to other phages when queried against the NCBI *Caudoviricetes* database (BLASTn query coverage, <10%; identity, <80%). In addition, unlike other T7-like phages infecting P. aeruginosa, the LUZ100 genome encodes an integrase gene (tyrosine site-specific recombinase) upstream of the RNA polymerase gene, suggesting that this phage could lysogenize its host cells (14, 15).

10.1128/msystems.01189-22.4 TABLE S4 LUZ100 coding sequences. Sequence similarity to T7 homologues is indicated in green, while similarity to other BLASTp hits is represented in black. Download Table S4, PDF file, 0.2 MB. Copyright © 2023 Putzeys et al. 2023 Putzeys et al. https://creativecommons.org/licenses/by/4.0/ This content is distributed under the terms of the Creative Commons Attribution 4.0 International license.

### (ii) Transcriptional landscape of LUZ100.

To gain more insights into the transcriptional scheme and gene regulation mechanisms of T7-like phages with temperate elements, we studied the viral transcriptome of LUZ100 using ONT-cappable-seq. In contrast to classic RNA sequencing approaches, ONT-cappable-seq allows end-to-end sequencing of primary transcripts and the identification of key regulatory elements in dense phage transcriptomes, such as transcription initiation and termination sites, as well as operon structures (16). To explore the phage transcriptome in a global fashion, RNAs from multiple time points throughout LUZ100 infection of its P. aeruginosa PaLo41 host were pooled and sequenced on a MinION sequencing device.

ONT-cappable-seq data analysis, followed by manual curation, revealed a total of 11 viral transcription start sites (TSSs) and 22 transcription termination sites (TTSs), together with their associated promoter (Table 1) and terminator (Table S5) sequences, across the LUZ100 genome (Fig. 2). Among the transcription termination sequences, five are predicted to be intrinsic, factor-independent terminators. Notably, many of the regulatory elements in LUZ100 are in conserved positions relative to T7, suggesting that the transcriptional scheme of LUZ100 is coordinated in a manner similar to that of the type coliphage.

**TABLE 1:**
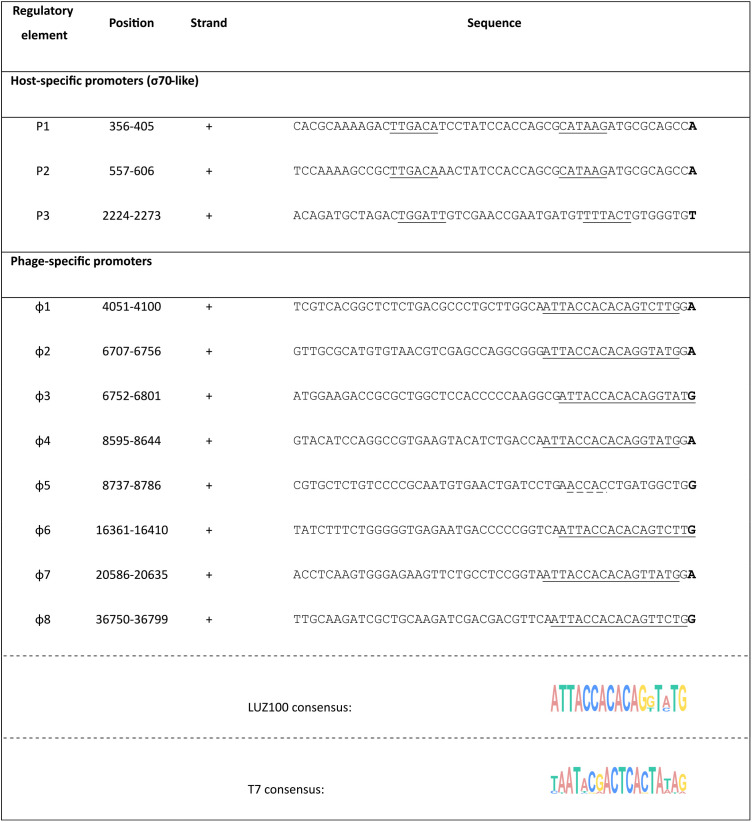
Overview of LUZ100 promoter elements identified by ONT-cappable-seq^*a*^

a TSSs are indicated in bold. For host-specific phage promoters, the σ^70^-like sequences are underlined. For the phage-specific promoters, a consensus motif was found using MEME (v 5.4.1) and is marked with a black line. Phage-promoter sequences that partly resemble the LUZ100 consensus motif are marked with a dotted line.

10.1128/msystems.01189-22.5 TABLE S5 LUZ100 terminators identified by ONT-cappable-seq. TTSs are indicated in bold. Intrinsic, factor-independent terminator sequences predicted by ARNold are indicated in blue. Their characteristic stem-loop structure is marked by the blue DNA sequence (stem) surrounding the underlined bases (loop). Download Table S5, PDF file, 0.1 MB. Copyright © 2023 Putzeys et al. 2023 Putzeys et al. https://creativecommons.org/licenses/by/4.0/ This content is distributed under the terms of the Creative Commons Attribution 4.0 International license.

As in most *Autographiviridae* phages, the early genomic region of LUZ100 encodes a suite of hypothetical proteins that are presumably involved in the host takeover process. In this region, we identified three TSSs and their cognate promoter sequences that show significant similarity to the σ^70^ consensus sequence of Pseudomonas. Upon infection, these host-specific promoters (P1, P2, and P3) are likely to be recognized by the bacterial RNAP and drive the expression of early genes. Interestingly, promoter P3, which drives transcription of the putative lysogeny gene module of LUZ100, deviates from the strongly conserved σ^70^ sequences of promoter P1 and P2. In addition, we observed that the majority of transcripts that start at P3 are strongly terminated by terminator T4, located directly downstream of the integrase gene. These findings suggest that transcription of the lysogeny-associated genes is regulated in a distinct manner independent from the other early genes, which is explored in more depth below.

The end of the early genomic region encodes the DNA-directed RNA polymerase. This RNAP shows high sequence similarity to the well-studied T7 RNAP (BLASTp E value of 9e−173). However, the LUZ100 RNAP domains that specifically recognize and bind the phage promoter sequences are only remotely related to the corresponding RNAP regions of phage T7 and various T7-like Pseudomonas phages (Table S6) (27, 28). This hints at an altered promoter specificity between the LUZ100 RNAP and the RNAP of T7 group members that infect Pseudomonas hosts. Based on the ONT-cappable-seq data, we identified five additional promoter sequences that are likely to be specifically recognized by the LUZ100 RNAP. These promoters share a highly conserved 17-bp motif (E value = 4.7 × 10^−10^) that only partially resembles the T7 consensus promoter (5′-TAATACGACTCACTATA**G**) (Table 1) (29). This motif was used to manually recover three additional promoters from the transcriptomic data that were not included previously, as they did not meet the stringent TSS threshold value. This yielded a total of eight phage-specific promoter sequences with a distinctive motif, further supporting the hypothesized orthogonality between different T7-like phage RNAP-promoter pairs (30).

10.1128/msystems.01189-22.6 TABLE S6 Alignment of the amino acid sequences of T7-like phage RNAPs involved in recognition and binding to promoter sequences. Download Table S6, PDF file, 0.1 MB. Copyright © 2023 Putzeys et al. 2023 Putzeys et al. https://creativecommons.org/licenses/by/4.0/ This content is distributed under the terms of the Creative Commons Attribution 4.0 International license.

The middle and late genes, which are transcribed by the phage RNA polymerase from their cognate phage promoters, are mainly responsible for phage DNA metabolism and virion morphogenesis, respectively (26). LUZ100 is equipped with the hallmark T7-like replication machinery, including a predicted single-stranded DNA binding protein, an endonuclease, a DNA polymerase, a DNA primase/helicase, and an exonuclease. In addition, the phage encodes a lysozyme-like protein that resembles the well-studied T7 lysozyme, which is involved in host cell lysis and inhibition of T7 RNAP transcription activity (BLASTp E value of 3e−36) (31, 32). In LUZ100, expression of genes involved in DNA metabolism appears to be largely driven by phage promoters ϕ2, ϕ3, ϕ4, and ϕ5. Remarkably, promoters ϕ2 and ϕ3 are organized in tandem, directly upstream of an annotated single-stranded-DNA-binding (SSB) protein. A similar observation was made for N4-like Pseudomonas phage LUZ7, in which two promoters in tandem achieved extremely high expression levels of a key SSB protein, the transcriptional regulator Drc (33). These results suggest that tandem promoters might be a common theme in phages to drive the expression of SSB proteins, which are often required in high abundance.

In contrast to T7, the replication module of LUZ100 contains three additional genes that show homology to genes encoding a thymidylate synthase, a ribonucleoside-diphosphate reductase (RNR), and a nucleotide pyrophosphohydrolase-like protein of the MazG protein family. These genes were presumably acquired from the host by horizontal gene transfer (15). Interestingly, auxiliary metabolic genes (AMGs), including those for RNRs and thymidylate synthases, were also found in other T7-like temperate phage genomes and are generally thought to reinforce the host metabolism during the phage infection process (13, 15). Both RNRs and thymidylate synthases are known to be involved in DNA metabolism and can presumably facilitate phage replication in the infected cell by increasing deoxynucleotide biosynthesis (34, 35). In contrast, phage-encoded nucleotide pyrophosphohydrolases are hypothesized to play a role in the regulation of cellular (p)ppGpp levels to maximize infection efficiency (36, 37).

Finally, the late genomic region of LUZ100 mainly encodes proteins involved in virion structure, assembly, DNA packaging, and host cell lysis. The structural gene cassette of LUZ100 includes genes coding for the portal protein, the major capsid protein (MCP), the tail fiber protein, tail tubular proteins A and B, internal virion proteins, and the small and large terminase subunits. Similar to T7, gene expression of the LUZ100 MCP appears to be tightly controlled by a local phage-specific promoter and an apparent strong terminator sequence located immediately downstream of the MCP gene. It should be noted that no obvious T7 internal virion protein C (gp15) and D (gp16) equivalents could be detected based on amino acid sequence similarity. However, given the strict synteny of the structural modules of T7, gh-1, and LUZ100, the translated products of LUZ100 *gp46* and *gp47* are likely to be functionally related. The ONT-cappable-seq data also revealed interesting transcriptional activity downstream of the LUZ100 MCP. Part of the transcripts in the structural region of the phage were transcribed antisense. A tBLASTx search was performed to verify whether incomplete annotation could explain the presence of this unexpected transcriptional activity. However, no obvious protein coding sequences were identified, leading us to speculate that the antisense transcripts correspond to a noncoding antisense RNA molecule. As previously hypothesized for LIT1 and LUZ7, these antisense RNAs putatively have a regulatory role in expression of the structural proteins (16, 38).

At the end of the infection cycle, the newly synthesized phage progeny is released by lysing the host cell. In general, the lysis pathway of T7-like phages is largely mediated by three elements—lysozyme, holin (type II), and spanins (Rz/Rz1)—all targeting different layers of the bacterial cell envelope. The LUZ100 lysozyme and holin could be identified and are actively transcribed during infection. However, no apparent spanin gene equivalents could be annotated after screening of the phage open reading frames (ORFs) for membrane localization signals that mark the internally overlapping Rz/Rz1 gene pair, as observed in T7 relatives (39). It has been suggested that, under certain physiological conditions, the lack of spanins could impede phage-mediated lysis and subsequent progeny release from Gram-negative bacteria (40). However, under laboratory conditions, LUZ100 appears to successfully breach the cell barriers of its host.

### (iii) LUZ100 transcription unit architecture.

Besides pinpointing transcriptional landmarks, the ONT-cappable-seq data were also used to elucidate the transcription unit architecture of LUZ100. Based on adjacent pairs of TSSs and TTSs defined in this work, we identified 37 unique transcription units (TUs) that cover on average 3.6 genes (Table S7). This large number of TUs is likely to be the result of sequential read-through across different TTSs (16). In general, we found that the genes that are cotranscribed in a TU are functionally related, as expected. For instance, whereas TU4 is devoted to transcription of the lysogeny module, TU17 and TU31 span genes involved in DNA metabolism, and virion morphology, respectively. In addition, several TUs show significant overlap in terms of their gene content, suggesting that overlapping TUs provide an alternative mechanism to fine-tune the expression levels for specific genes. Similar observations have been made in bacteria, where complex TU clusters are thought to be employed as a regulatory strategy to modulate gene expression levels under different conditions (41–43).

10.1128/msystems.01189-22.7 TABLE S7 Transcriptional units of LUZ100. The transcriptional units are delineated by the TSSs and TTSs identified by ONT-cappable-seq. Download Table S7, PDF file, 0.4 MB. Copyright © 2023 Putzeys et al. 2023 Putzeys et al. https://creativecommons.org/licenses/by/4.0/ This content is distributed under the terms of the Creative Commons Attribution 4.0 International license.

### Hypothesized lysogeny of LUZ100.

Recently, multiple incidences of temperate T7-like phages have been reported, contradicting the general assumption that T7-like phages propagate according to a strictly lytic life cycle (14, 15, 44, 45). However, general phage characterization of LUZ100, in combination with genomic and transcriptomic analyses, revealed low virulence and an actively transcribed phage-encoded integrase. These traits are both clues that LUZ100 could be prone to a lysogenic life cycle and put LUZ100 forward as a new member of the emerging clade of T7-like temperate *Autographiviridae* phages.

**FIG 3:**
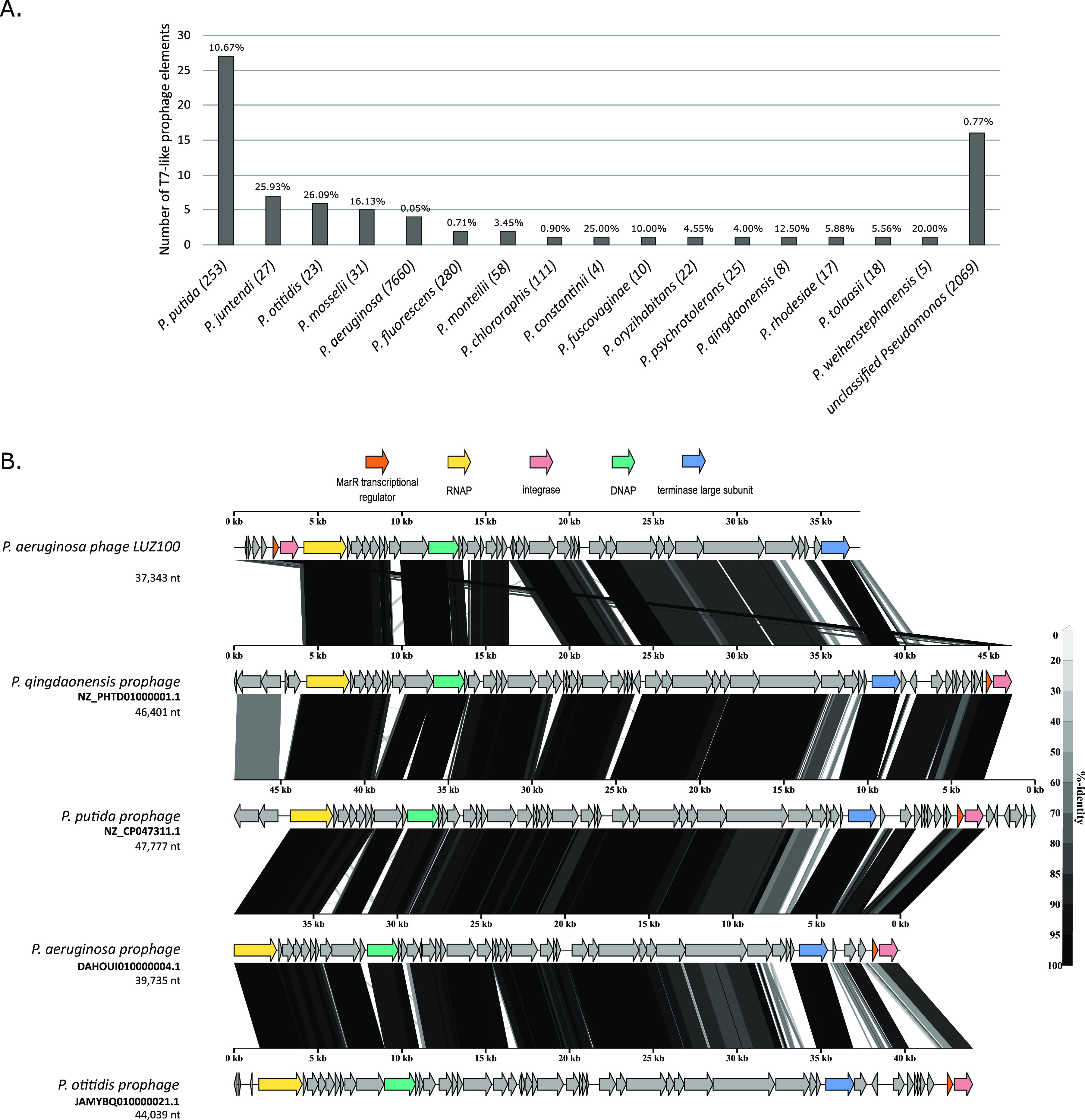
T7-like prophages in Pseudomonas genomes. (A) Bar plot showing the distribution of T7-like prophage elements identified in the genomes of different Pseudomonas species. For each species, the number of publicly available genomes on NCBI (accessed on 09 January 2023) is indicated in parentheses. (B) Genomic similarity of LUZ100 to T7-like prophages found in Pseudomonas. Alignment of the LUZ100 genome to four prophages retrieved from different Pseudomonas species. The image was generated using VipTree (59). The arrows represent the integrase (pink), the MarR transcriptional regulator (orange) the RNA polymerase (yellow), the DNA polymerase (green), and the terminase large subunit (blue). Identity, indicated in grayscale, represents the amino acid sequence similarity, based on a tBLASTx search.

Notably, T7-related prophage elements are widespread across the genomes of various Gram-negative bacterial species and, to a lesser extent, Gram-positive bacteria (14). The LUZ100 integrase also displays homology to the integrases from T7-like temperate phages Pasto (MT708545.1) and HTVC019P (NC_020483.1), which were shown to successfully integrate in the genomes of their hosts, *Agrobacterium* and *Pelagibacter*, respectively (BLASTp query coverage, >70%; identity, >32%; E value, <e−48) (14, 15). To further assess the distribution and conservation of T7-like prophage elements in Pseudomonas, we identified Pseudomonas strains that carry prophages similar to LUZ100. For this, a BLASTp search using the LUZ100 RNAP as a query was performed, which resulted in 78 Pseudomonas genomes that harbor T7-like prophage elements (Fig. 3A). Although the P. aeruginosa genomes are the most prevalent on NCBI (7,660 genomes; accessed 9 January 2023), this number of identified T7-like prophages is marginal (4 prophages [0.05%]). In contrast, less abundantly sequenced Pseudomonas species, such as P. putida (253 genomes; accessed 9 January 2023) and P. juntendi (27 genomes, accessed 09 January 2023), show a relatively higher proportion of T7-like prophages incorporated in their genomes, with 27 (10.7%) and 7 (25.9%) hits, respectively. However, more Pseudomonas genomes should be made available to evaluate which species are targeted more often by T7-like temperate phages. Furthermore, when several of the LUZ100 core genes, including the RNA and DNA polymerase, the integrase, and the terminase large subunit, were compared to homologs found in the T7-like Pseudomonas prophages, sequence identities up to 98.57% at the protein level were observed (Fig. 3B). These results, together with the wide distribution of T7-like prophage elements found across the genus Pseudomonas, strengthen the hypothesis that LUZ100 could also pursue a temperate replication strategy, albeit in rare cases. Another feature that points in the same direction is the presence of two tRNA genes on the LUZ100 genome. tRNAs are abundantly present in phage genomes. However, when virulent and temperate phages are compared, the former tend to have more copies (46). In addition to the low copy number of tRNAs encoded in the LUZ100 genome, one of the tRNA genes, encoding a tRNA-Leu, is located immediately upstream of the integrase gene. Since tRNAs are considered integrational hot spots for phages and other mobile genetic elements, this tRNA molecule might be recruited by imprecise excision after a previous integration event (47). A similar hypothesis was proposed for temperate T7-like *Pelagibacter* phages that also retained a tRNA-Leu sequence upstream of their integrase (15). The remnants of the previous integration event in combination with the low tRNA abundance in the LUZ100 genome are additional clues that link LUZ100 to a lysogenic life cycle.

As previously mentioned, transcriptome analysis revealed that the LUZ100 integrase gene is actively transcribed. Directly upstream of the integrase, LUZ100 encodes a MarR-like transcriptional regulator. This regulator has been identified in several integrase-coding T7-like phages and was recently proposed to be involved in their lysis/lysogeny decision (8, 14, 15). The ONT-cappable-seq data show that the MarR-like regulator and the integrase are cotranscribed in an operon from the host-specific promoter P3. Since this promoter deviates from the highly conserved σ^70^ sequences of promoter P1 and P2, a fluorescence expression assay was performed to validate the activity of the promoter *in vivo*. For this purpose, the P3 promoter was cloned upstream of a ribosomal binding site (RBS) and an *msfGFP* gene using the SEVAtile DNA assembly method. Subsequently, the resulting vector was transformed to the laboratory model organism Escherichia coli (48). The P3 promoter shows significantly (*P* < 0.001) higher expression of the fluorescent reporter than the negative control without promoter sequence, confirming its activity *in vivo* (Fig. S3). The transcriptional activity of both the MarR-like regulator and the integrase further supports our hypothesis that LUZ100 is prone to a lysogenic life cycle through MarR-based regulation.

10.1128/msystems.01189-22.10 FIG S3 *In vivo* experimental validation of promoter P3 in E. coli. The promoter activity was determined by measuring the levels of msfGFP. They were normalized to OD_600_ and converted to absolute values using 5(6)-FAM as a calibrant [represented by the 5(6)-FAM/OD_600_ axis]. The negative control (NC) represents a pBGDes vector lacking a promoter (pBGDes BCD2-msfGFP), while the positive control (PC) shows the results for a vector containing a constitutive promoter (pBGDes Pem7-BCD2-msfGFP). The asterisk indicates a significant difference of the promoter in comparison to the NC (based on a Dunnett test; *P* < 0.001). Data are means for three biological replicates, and standard deviations are indicated with error bars. Download FIG S3, PDF file, 0.05 MB. Copyright © 2023 Putzeys et al. 2023 Putzeys et al. https://creativecommons.org/licenses/by/4.0/ This content is distributed under the terms of the Creative Commons Attribution 4.0 International license.

**FIG 4:**
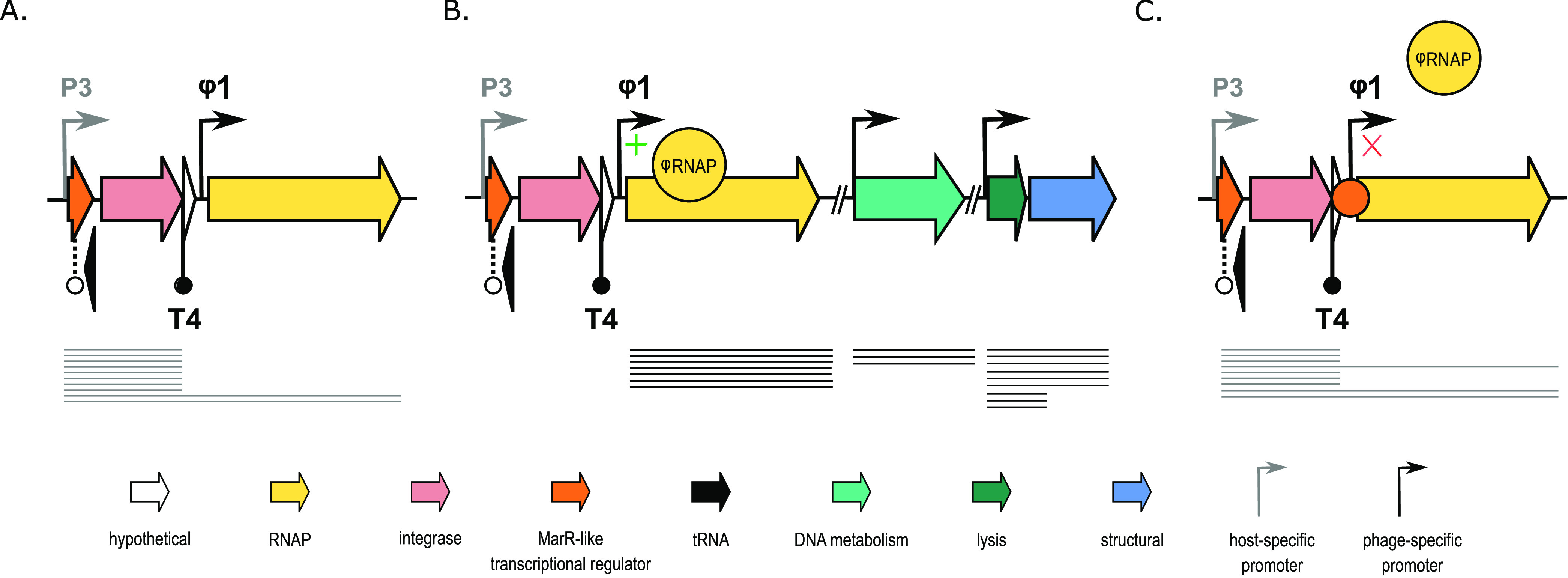
Schematic representation of the hypothesized MarR-based lysogeny control mechanism of LUZ100. (A) Schematic of the putative MarR-based lysogeny control region of LUZ100. Upon infection, the host RNAP transcribes the lysogeny module, including the MarR-like protein and integrase. A limited number of these transcripts read through T4 and transcribe the phage RNAP. (B) Once expressed, the phage RNAP can initiate transcription from phage-specific promoter ϕ1 and amplify its own transcription. Sufficient expression of the phage RNAP enables expression of the LUZ100 middle and late genes from phage-specific promoters, which are required to complete the lytic infection cycle. (C) Lysogeny is established by binding of the MarR-like repressor protein to specific binding sites that inhibit transcription initiation from ϕ1 by the phage RNAP.

The phage RNAP is key to complete a lytic infection cycle, and the regulation of the polymerase is proposed to be intertwined with the MarR-based transcriptional regulation in some of the temperate T7-like phages (14). During lysogeny, the MarR regulator is hypothesized to repress phage RNAP expression through recognition of specific binding sites sequences that flank the promoter sequence upstream of the RNAP (14). Interestingly, in case of LUZ100, the location of phage-specific promoter ϕ1, encoded directly upstream of the RNAP, raises questions on how the RNAP becomes initially activated. Visual inspection of the long-read transcriptional landscape of the lysogeny cassette and RNAP of LUZ100 revealed that not all transcripts are terminated after the integrase gene. Based on these findings, we hypothesize a lysogeny control mechanism where the host RNAP sporadically reads through terminator T4 and transcribes the phage RNAP from the host-specific promoter P3, located further upstream (Fig. 4A). Once the phage RNAP is transcribed by the host RNAP and expressed, it is able to bind the phage-specific promoter and induce its own transcription in a positive feedback loop (Fig. 4B). In contrast, when the lysogenic state is favored, transcription from the phage-specific promoter is repressed by the MarR-like protein, and middle and late gene expression is impaired (Fig. 4C). However, it should be noted that our data set did not capture any sufficiently long reads starting at promoter P3 that span the RNAP entirely, which might be attributed to the limited sequencing depth and technical limitations of ONT-cappable-seq (16).

As stated previously, stable lysogen formation could not be confirmed in several susceptible P. aeruginosa strains in our panel (Fig. S1). The apparent lack of established lysogeny in these host might be explained by the absence of appropriate genome integration sites or the lack of specific bacterial regulatory elements that favor MarR-based repression and hence prophage stability. Alternatively, it cannot be excluded that the MarR binding sequences in the lysis-lysogeny switch in LUZ100 might be defective, and the phage has (partially) lost its lysogenic capacity over the course of evolution. As the hypothesis of an ancient temperate origin of strictly virulent T7-like phages is becoming more apparent, it is tempting to speculate that LUZ100 represents an evolutionary intermediate between the temperate lineage and the strictly virulent T7 descendants (14).

### Conclusion and perspectives.

The enormous diversity of bacteriophages is again highlighted in our analysis of a novel phage infecting P. aeruginosa, LUZ100. In contrast to the strictly lytic model phage T7, a distant relative in the *Autographiviridae* family, LUZ100 contains key genes associated with temperate behavior. Recently, a number of T7-like phages that show characteristics similar to those of LUZ100 were identified (14, 15, 44). However, concrete evidence of entering and maintaining the lysogenic life cycle has remained ambiguous for these phages (14, 15, 44). Also in our research, no stable lysogens have been obtained to date.

To this end, transcriptomics-driven characterization of bacteriophage LUZ100 was performed to study the molecular processes at work in this peculiar member of the *Autographiviridae* (16). The ONT-cappable-seq data showed active cotranscription of the MarR-like transcriptional regulator and the integrase from the host-specific promoter P3, which further strengthened the hypothesis that not all T7-like phages should automatically be assumed to have a strictly lytic life cycle. Moreover, the identification of a phage-specific promoter driving transcription of the phage-encoded RNA polymerase suggests that the regulation of this polymerase is interwoven with the activity of the MarR-based regulator and likely plays a role in the lysis/lysogeny switch of LUZ100.

In addition to our findings, others have speculated that from an evolutionary perspective, T7-like phages could be descendants from a common temperate ancestor that (partly) lost its ability to lysogenize its host over time (14). This has several consequences for fields like phage therapy, where virulent phages are preferred for therapeutic purposes (49). Consistently screening for characteristics associated with a lysogenic life cycle is required to put forward phages suitable for phage therapy. As shown in this work, the combination of multiple omics techniques can serve this purpose. Whereas DNA sequencing can identify the presence of lysogeny-related genes, RNA sequencing approaches can give information on their transcriptional activity and underlying regulatory mechanisms.

The ONT-cappable-seq data allowed us to identify key transcriptional elements of LUZ100, including TSSs, TTSs, and TUs. The RNAP-promoter pairs identified in this work are distinct from those of other T7-like phages and could be further explored for synthetic biology applications. Furthermore, other peculiar transcriptional features, such as the presence of antisense RNA and a phage-specific promoter in front of the phage RNAP, show that our knowledge on the molecular diversity of phages overall remains limited. However, the ONT-cappable-seq approach has the potential to help bridge these knowledge gaps by generating a bird’s-eye view of phage transcriptomes in an efficient yet cost-effective manner.

## MATERIALS AND METHODS

### Bacterial strains and culture conditions.

The collection of P. aeruginosa strains in this study includes laboratory strains PAO1k, PA7, and PA14, 24 strains from the Pirnay collection, and 47 clinical strains that were isolated from the respiratory tracts of cystic fibrosis (CF) patients in the Leuven University Hospital, Leuven, Belgium (50). Bacteria were cultivated in lysogeny broth (LB) liquid medium with shaking or plated onto LB agar (1.5% [wt/vol]) at an incubation temperature of 37°C.

### Phage isolation, propagation, and purification.

Clinical P. aeruginosa strain PaLo41 was used for phage isolation and propagation. Phage LUZ100 was isolated from a sewage sample of the Leuven University Hospital, Leuven, Belgium. The sewage sample was centrifuged at 4,000 × *g* for 30 min and filtered using a 0.45-μm filter to remove bacteria and environmental debris. A 5-mL aliquot of filtered water sample was mixed with an equal volume of 2× LB liquid medium, 100 μL of exponentially growing bacterial culture, and 1 mM CaCl_2_ and cultured at 37°C overnight with constant shaking. After adding a few drops of chloroform and centrifugation for 1 h at 4,000 × *g*, the supernatant was filtered again through a 0.45-μm filter and screened for the presence of phages according to the standard double-agar overlay method using a 0.5% LB agar top layer (51). Single plaques were picked up to start a next round of propagation. This process was repeated several times to produce a homogenous phage stock. Next, the phage was amplified on plates by performing five agar overlay assays in parallel and overnight incubation at 37°C. The lysed top layers were collected in a tube, complemented with 30 mL of phage buffer (8.77 g NaCl, 2.47 g MgSO_4,_ 1.21 g Tris-HCl [pH 7.5] in 1 L of distilled H_2_O) and chloroform (1% [vol/vol]) and vigorously shaken overnight. Next, the solution was centrifuged for 40 min at 4,000 × *g* and filtered (0.45 μm) to remove residual bacterial debris. The crude phage lysate was concentrated and purified by polyethylene glycol 8000 (PEG 8000) precipitation as previously described by Ceyssens et al. (52), with minor modifications. Briefly, phages were precipitated overnight at 4°C, spun down at 4,000 × *g* for 40 min, resuspended in 3 mL phage buffer, and stored at 4°C for further analysis.

### Host range analysis.

The host range of phage LUZ100 against our characterized collection of P. aeruginosa strains was determined by spotting 1.5 μL of 1:10 dilutions of the phage stock on an initiated bacterial lawn of each strain, created with a double agar overlay using a 0.5% LB agar top layer. After overnight incubation at 37°C, the success of phage infection was assessed by surveying the clearance of the spots in the bacterial lawn, and given a score of 1 for complete lysis, 2 for lysis with individual plaques, and 3 for no lysis. The bacterial strains were considered susceptible to the phage only when distinct plaques could be observed, as clearing zones could also arise due to lysis effects that do not rely on productive phage infection (53).

### Adsorption assay.

To assess the adsorption kinetics of LUZ100 to P. aeruginosa PaLo41, an adsorption assay was performed using three biological replicates. PaLo41 was grown in LB medium supplemented with 1 mM CaCl_2_ (Sigma-Aldrich) and 1 mM MgCl_2_ (Sigma-Aldrich) and grown to the early exponential phase (optical density at 600 nm [OD_600_] = 0.3). At this point, a sample was taken and plated to determine the bacterial titer (*B*). Next, the bacteria were infected with LUZ100 at an MOI of 0.01 and incubated at 37°C. Subsequently, 100-μL samples were taken 1, 5, 10, 15, and 20 min (*t*) postinfection and directly transferred to an Eppendorf tube with an excess of chloroform to kill the bacteria. For each time point sample, the phage titer (*P*) was determined using the double agar overlay method. Finally, the average phage adsorption constant was calculated using the following formula (51):

$$k=\frac{2.3}{\mathrm{Bt}}\times \;\log(\frac{P}{P_{0}})$$

### Infection curve.

The bacteriolytic activity of phage LUZ100 was determined by monitoring the growth of the phage-infected bacteria over time. For this, overnight cultures of three biological replicates of PaLo41 were inoculated in fresh LB medium and incubated at 37°C to an OD_600_ of 0.3. Next, these cultures were infected with LUZ100 at an MOI of either 1 or 10. The OD_600_ of the uninfected and infected cultures was measured every 15 min for 145 min on the CLARIOstar Plus microplate reader (BMG Labtech, Ortenberg, Germany) for four technical replicates during incubation at 37°C. To assess the lytic activity of LUZ100, the phage score (*P_S_*) and virulence index (*V*_ϕ_) were determined (19, 20). For this, the area under the infection curve (*A*) was determined using the statistical software JMP. The following formulas were applied, for which the virulence curve represents a plot of *v*_MOI_ as a function of the MOI (NC = negative control):

$$P_{s}=\frac{\sum_{i=1}^{n}\frac{A_{\text{MOI }(i)}/A_{\mathrm{NC}}}{\text{MOI }(i)}}{\sum_{i=1}^{n}\frac{1}{\text{MOI }(i)}}$$

$$v_{\text{MOI}}=1-\frac{A_{\text{MOI}}}{A_{\text{NC}}}$$

$$V\phi =\frac{A_{\text{virulence curve}}}{A_{\text{max}}}$$

### Transmission electron microscopy.

Transmission electron microscopy (TEM) images were made as described by Vallino et al. (54). Briefly, a phage suspension was adsorbed onto carbon and copper-palladium grids coated with Formvar for 3 min. Next, the grids were rinsed with water and negatively stained with 0.5% aqueous uranyl acetate. Samples were visualized using a CM10 transmission electron microscope (Philips, Eindhoven, The Netherlands) at a voltage of 80 kV.

### LUZ100 receptor analysis.

To identify the LUZ100 receptor(s), the genomic DNA (gDNA) of four spontaneous phage-resistant PaLo41 colonies was isolated using the Qiagen DNeasy Ultraclean microbial kit according to the manufacturer’s guidelines. The gDNA samples were prepared using the Nextera DNA Flex library preparation kit, and paired-end sequencing was performed on an Illumina MiniSeq device. Next, the quality of the reads was assessed with FastQC (v0.11.8) (55), and the adapters and poor-quality reads (Phred score < 33) were removed from the data set using Trimmomatic (v0.39) (56). Finally, Snippy (v4.6.0) (https://github.com/tseemann/snippy) was used to identify SNPs in the genomes of the LUZ100-resistant PaLo41 clones compared to the PaLo41 reference genome (BioProject no. PRJNA731114).

### Identification of prophages similar to LUZ100 in Pseudomonas.

To identify prophages related to LUZ100, a BLASTp search of the nonredundant protein sequences (nr) database filtered for organism Pseudomonas (taxid 286) was performed (accessed 09 January 2023) (57). For this, the LUZ100 RNA polymerase was used as a query. Next, the resulting Pseudomonas genomes were uploaded in PHASTER to identify and extract the prophage region, followed by manual curation (58). Finally, the prophages with the highest homology to the LUZ100 RNAP (BLASTp query coverage, >95%; identity, >95%) were aligned to LUZ100 to determine genomic similarity using ViPTree (59).

### Lysogen generation.

Based on LUZ100 host range and infection efficiency, six phylogenetically distinct P. aeruginosa strains were selected to isolate putative LUZ100 lysogens: PaLo41, PaLo44, PaLo226, PaLo249, PaLo287, and PaLo402. For this, 20 μL of a high-titer phage stock was spotted on an initiated bacterial lawn of the different strains using a double agar overlay with a 0.5% top layer of LB agar. After overnight incubation at 37°C, resistant colonies in the clearing zones were picked and propagated on fresh LB agar plates three consecutive times to exclude carryover of phage particles. Next, 16 individual colonies of each P. aeruginosa strain (14 for strain PaLo402) were screened for the presence of LUZ100 by PCR with phage-specific primers targeting a fraction of the phage RNA polymerase (Table S1; Fig. S1).

10.1128/msystems.01189-22.1 TABLE S1 Primers, inserts, and vectors used in this study. Download Table S1, PDF file, 0.10 MB. Copyright © 2023 Putzeys et al. 2023 Putzeys et al. https://creativecommons.org/licenses/by/4.0/ This content is distributed under the terms of the Creative Commons Attribution 4.0 International license.

### LUZ100 genome extraction, sequencing, and genomic analysis.

LUZ100 phage lysate was subjected to DNase I (Thermo Fisher Scientific) and RNase A (Thermo Fisher Scientific) treatment for 1 h in a 37°C water bath. The lysate was treated with sodium dodecyl sulfate (SDS) (Acros Organics), EDTA (Sigma-Aldrich), and proteinase K (Thermo Fisher Scientific) and incubated for 1 h in a 56°C water bath. Next, the DNA of LUZ100 was isolated by phenol-chloroform extraction and subsequently purified by ethanol precipitation. The purity and concentration of the DNA were assessed on a SimpliNano spectrophotometer (Biochrom US Inc.) and a Qubit 4 fluorometer (Thermo Scientific), respectively. Library preparation, Illumina sequencing, and raw read processing were performed as described above. The genomic sequence of LUZ100 was assembled with SPAdes (v3.13.2) using default parameters (60), followed by visual inspection of the assembly in Bandage (61). In addition, the phage genome was sequenced in full length using nanopore sequencing to identify the genomic termini. For this, the phage DNA was prepared with a rapid barcoding kit (SQK-RBK004) (Oxford Nanopore Technologies) and sequenced on a MinION device (FLO-MIN 106, R9.4). The raw sequencing data were base-called using Guppy (v3.4.4), and reads were cleaned with Porechop (v0.2.3). The phage genome was assembled using Unicycler (v0.4.8) (62) and subsequently annotated using the phage-specific RAST pipeline in PATRIC (v3.6.1) (63–65). Manual curation was performed by scanning each predicted coding sequence for homologs using HMMR, BLASTp, and HHPred with default settings, as provided by the MPI Bioinformatics Toolkit (66). This annotation was subsequently used for transcriptomic data analysis.

### RNA extraction, sequencing and transcriptomic analysis. (i) RNA sampling and extraction.

An overnight culture of PaLo41 was diluted 1:100 in 50 mL LB medium, incubated at 37°C, and grown to an OD_600_ of 0.3. At this point, a 4.5-mL sample was mixed with 0.9 mL stop mix solution (95% [vol/vol] ethanol, 5% [vol/vol] phenol; saturated, pH 4.5) and immediately snap-frozen in liquid nitrogen (sample *t*_0_). The remaining culture was infected with LUZ100 (MOI = 10) and incubated at 37°C. Additional samples were taken every 4 min up to 40 min after infection. Samples were thawed on ice, pelleted by centrifugation (20 min, 4°C, 4,500 rpm) and resuspended in a 0.5-mg/mL lysozyme solution. Subsequently, all samples, aside from *t*_0_, were pooled in equal amounts and homogenized (sample *t*_ϕ_). The resulting samples *t*_0_ (uninfected) and *t*_ϕ_ (phage infected) were subjected to hot phenol treatment and ethanol precipitation to extract and purify the RNA. Next, *t*_0_ and *t*_ϕ_ were treated with DNase I and subsequently purified using ethanol precipitation and spin column purification. The absence of genomic DNA was verified by PCR using a phage-specific and host-specific primer pair (Table S1). Finally, the integrity of the RNA samples was evaluated by running the samples on an Agilent 2100 Bioanalyzer using the RNA 6000 Pico kit. Samples with an RNA integrity number (RIN) of ≥9 were used for downstream processing and sequencing.

### ONT-cappable-seq and data analysis.

Prokaryotic RNA samples *t*_0_ and *t*_ϕ_ were supplemented with 1 ng of an *in vitro*-transcribed control RNA spike-in (1.8 kb), which was synthesized using the HiScribe T7 high-yield RNA synthesis kit according to the manufacturer’s guidelines (New England Biolabs). Next, library preparation of the RNA samples was performed according to the ONT-cappable-seq method (16). Equimolar amounts of the resulting *t*_0,enriched_, *t*_0,control_, *t*_ϕ,enriched_, and *t*_ϕ,control_ cDNA samples were pooled in a 10-μL volume. The final library was loaded on a MinION flow cell (FLO-MIN 106, R9.4) and sequenced using the MinION platform for >48 h until all pores were exhausted. In parallel, the MinIT device with build-in Guppy software (v3.2.10) was used in high-accuracy mode to simultaneously base-call and demultiplex the reads, retaining only the reads with sufficient quality (>7). The overall performance of the sequencing run and raw read quality were assessed using NanoComp (v1.11.2). Next, raw reads were processed and subsequently mapped to the genomes of Pseudomonas phage LUZ100 and P. aeruginosa host strain PaLo41, as described previously (16). Sequencing quality, read lengths, and mapping metrics are reported in Table S2. Alignments were visualized in Integrative Genomics Viewer (IGV) (67). Finally, data analysis was performed according to the ONT-cappable-seq workflow (https://github.com/LoGT-KULeuven/ONT-cappable-seq) to identify viral TSSs and TTSs and elucidate the transcriptional architecture of LUZ100 (16).

10.1128/msystems.01189-22.2 TABLE S2 Sequencing metric. (A) Raw reads. (B) Processed reads. (C) Mapped reads. Download Table S2, PDF file, 0.1 MB. Copyright © 2023 Putzeys et al. 2023 Putzeys et al. https://creativecommons.org/licenses/by/4.0/ This content is distributed under the terms of the Creative Commons Attribution 4.0 International license.

To discriminate between phage-specific and host-specific promoter sequences, regions upstream of the annotated TSSs (−100 to +1) were analyzed using MEME and the Pseudomonas σ^70^ promoter prediction tool SAPPHIRE.CNN (68, 69). The motif of the identified phage-specific promoter sequences was used to conduct a MAST search on the LUZ100 genome to detect TSSs originally missed in the ONT-cappable-seq workflow (70). Based on the MAST results and manual inspection of the LUZ100 transcriptional landscape, three additional phage promoters were added to the list of regulatory elements. In parallel, for each TTS identified with ONT-cappable-seq, the surrounding region from −60 to +40 was uploaded in ARNold to predict intrinsic, factor-independent transcription termination sequences (71). The TUs of LUZ100 were defined by neighboring TSSs and TTSs determined in this study, if supported by ONT-cappable-seq reads that span the candidate TU. In cases where transcripts from a specific TSS lacked a defined TTS, the longest transcript was used to delineate the TU. Genetic features located on the same strand were annotated into a TU if they were covered by at least 90% (bedtools intersect -F 0.9 -s). Based on the TUs and their gene content, complex operon structures were delineated by finding overlapping TUs with the same orientation that share at least one annotated gene (16, 72).

### *In vivo* promoter activity assay.

To validate the activity of host-specific promoter P3 *in vivo*, the SEVAtile DNA assembly method was used to clone the promoter upstream of a standardized ribosomal binding site variant (BCD2v) and an *msfGFP* gene (48). The construct was transformed to E. coli *pir2* cells, which were plated on LB agar containing kanamycin (50 μg/mL). A pBGDes vector lacking a promoter (pBGDes BCD2v-msfGFP) and a vector containing a constitutive promoter (pBGDes Pem7-BCD2v-msfGFP) were used as a negative and positive control, respectively. The used vectors, primers, and tiles are listed in Table S1. Next, three biological replicates of the transformed E. coli cells were inoculated in M9 medium (1× M9 salts [BD Biosciences], 0.5% casein amino acids [LabM; Neogen], 2 mM MgSO_4_, 0.1 mM CaCl_2_ [Sigma-Aldrich], 0.2% citrate [Sigma-Aldrich]) complemented with 50 μg/mL kanamycin. The next day, cultures were diluted in fresh M9 medium, transferred to a Corning 96-well black polystyrene microplate with a clear flat bottom, and incubated with shaking. After 3.5 h, the OD_600_ and levels of monomeric superfolder green fluorescent protein (msfGFP) were measured on a CLARIOstar multimode plate reader (BMG Labtech, Ortenberg, Germany). Then, the msfGFP levels were normalized for OD_600_ and converted to absolute units using 5(6)-carboxyfluorescein [5(6)-FAM] (Sigma-Aldrich) as a calibrant (73). Finally, the statistical software JMP was used to analyze the data (74).

### Data availability.

The genomes of P. aeruginosa strain PaLo41 and phage LUZ100 were deposited in NCBI GenBank (BioProject accession numbers PRJNA731114 and PRJNA870687, respectively). Raw RNA sequencing files were deposited under GEO accession number GSE211961. All scripts and codes used in this study are available on GitHub (https://github.com/LoGT-KULeuven/ONT-cappable-seq). Any additional information is accessible from the authors upon request.
