# Supplementary figures and images for: Transcriptomics-Driven Characterization of LUZ100, a T7-like Pseudomonas Phage with Temperate Features
Source: mSystems. 2023 Feb 16;8(2):e01189-22. doi: 10.1128/msystems.01189-22 (PMC10134795; doi:10.1128/msystems.01189-22)

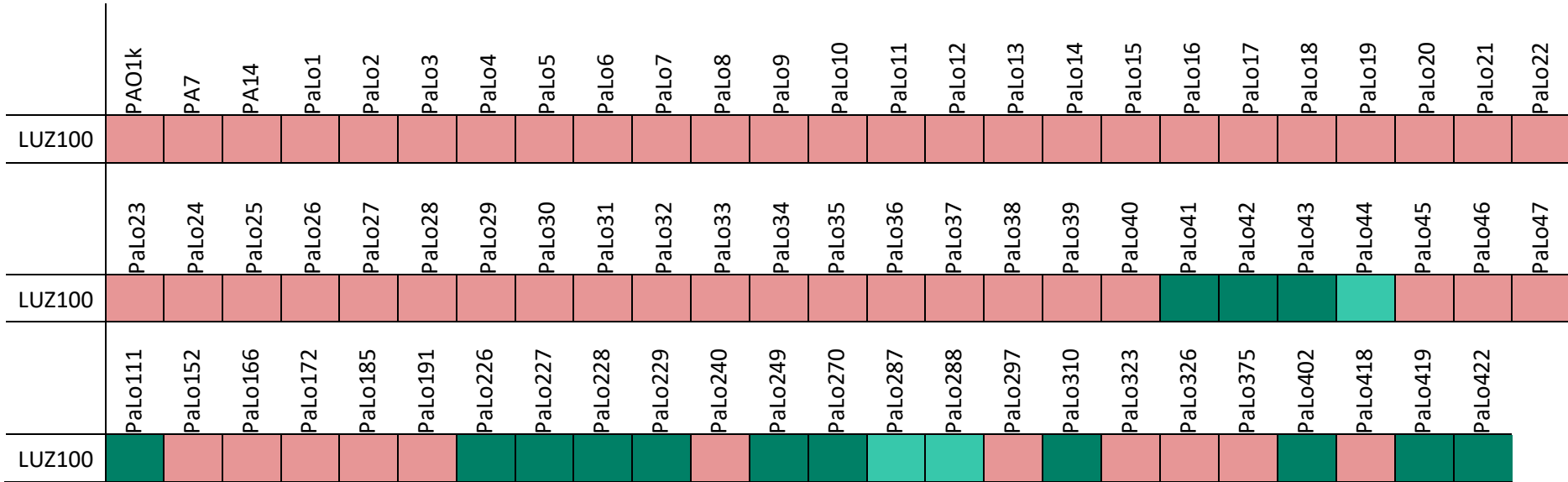

Supplement: FIG S2 [file msystems.01189-22-s0002.pdf]

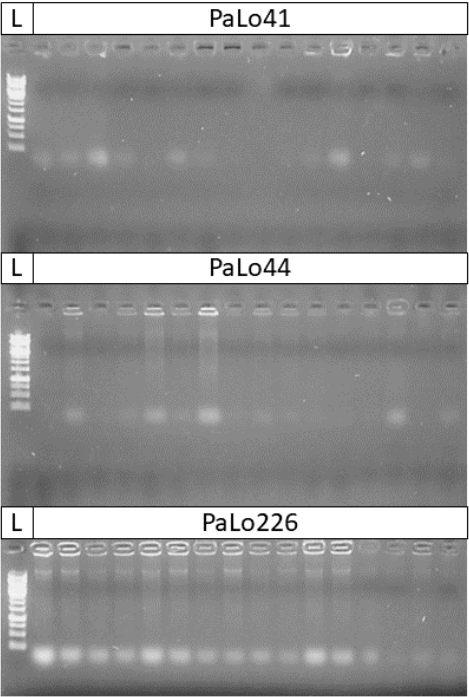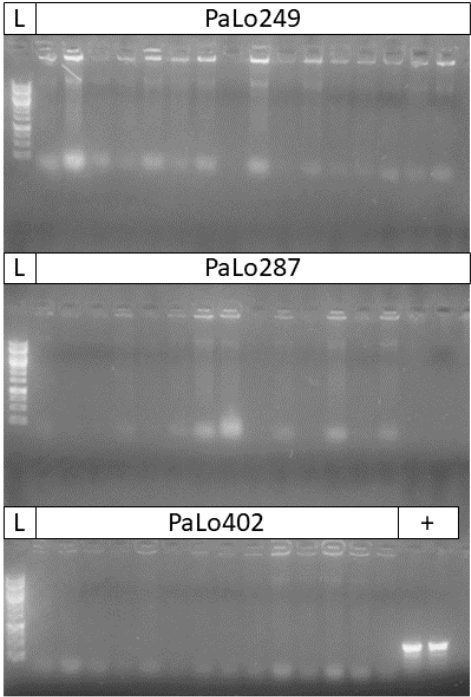

Supplement: FIG S1 [file msystems.01189-22-s0001.pdf]

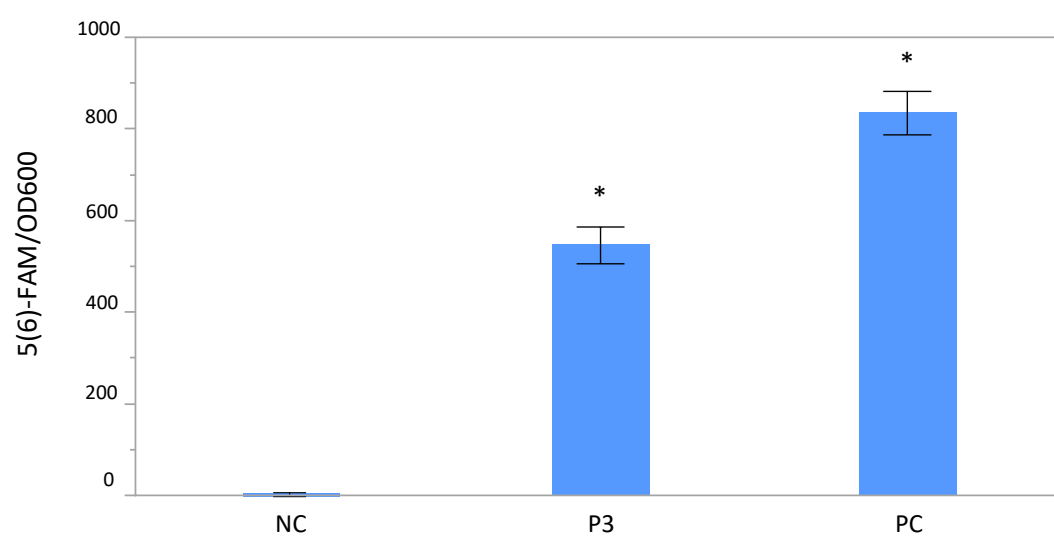

Supplement: FIG S3 [file msystems.01189-22-s0010.pdf]
