# Supplementary material for: Transcriptomics-Driven Characterization of LUZ100, a T7-like Pseudomonas Phage with Temperate Features
Source: mSystems. 2023 Feb 16;8(2):e01189-22. doi: 10.1128/msystems.01189-22 (PMC10134795; doi:10.1128/msystems.01189-22)
Supplement: TABLE S3 [file msystems.01189-22-s0005.pdf]

**Supplementary Table S3**

| Replicate | Position | Type* | Reference | Resistant strain | Palo41 gene |
|---|---|---|---|---|---|
| 1 | 70507 | deletion | AG | A | Intergenic region |
| 1 | 951537 | insertion | C | CG | Hypothetical protein |
| 1 | 958920 | single nucleotide polymorphism | C | T | Putative diguanylate cyclase DgcE |
| 1 | 1965539 | complex | AC | G | 5-oxoprolinase subunit C |
| 1 | 1965550 | insertion | C | CG | 5-oxoprolinase subunit C |
| 1 | 1965635 | single nucleotide polymorphism | A | G | 5-oxoprolinase subunit C |
| 1 | 1966370 | deletion | AG | A | 5-oxoprolinase subunit A |
| 1 | 2188204 | single nucleotide polymorphism | A | G | Intergenic region |
| 1 | 2828940 | single nucleotide polymorphism | T | C | Translation initiation factor IF-3 |
| 1 | 3094356 | single nucleotide polymorphism | G | A | Transcription-repair-coupling factor |
| 1 | 3462822 | insertion | G | GC | Hypothetical protein |
| 1 | 3549107 | insertion | G | GC | Intergenic region |
| 1 | 3822391 | insertion | C | CT | Intergenic region |
| 1 | 3852621 | single nucleotide polymorphism | G | A | cheB_2 |
| 1 | 4049509 | single nucleotide polymorphism | G | A | Respiratory nitrate reductase 2 beta chain |
| 1 | 4125656 | insertion | G | GC | Intergenic region |
| 1 | 4289904 | single nucleotide polymorphism | C | A | Sorbitol dehydrogenase |
| 1 | 4432563 | insertion | C | CG | Hypothetical protein |
| 1 | 4516675 | single nucleotide polymorphism | T | C | 3-demethoxyubiquinol 3-hydroxylase |
| 1 | 5865545 | deletion | AG | A | O-antigen ligase WaaL |
| 2 | 70507 | deletion | AG | A | Intergenic region |
| 2 | 951537 | insertion | C | CG | Hypothetical protein |
| 2 | 958920 | single nucleotide polymorphism | C | T | Putative diguanylate cyclase DgcE |
| 2 | 1180554 | single nucleotide polymorphism | G | T | Hypothetical protein |
| 2 | 1965539 | complex | AC | G | 5-oxoprolinase subunit C |
| 2 | 1965550 | insertion | C | CG | 5-oxoprolinase subunit C |
| 2 | 2188204 | single nucleotide polymorphism | A | G | Intergenic region |
| 2 | 2828940 | single nucleotide polymorphism | T | C | Translation initiation factor IF-3 |
| 2 | 3094356 | single nucleotide polymorphism | G | A | Transcription-repair-coupling factor |
| 2 | 3124795 | single nucleotide polymorphism | A | G | Molybdenum cofactor guanylyltransferase |
| 2 | 3462822 | insertion | G | GC | Hypothetical protein |
| 2 | 3549107 | insertion | G | GC | Intergenic region |
| 2 | 3549117 | insertion | G | GC | Intergenic region |
| 2 | 4289904 | single nucleotide polymorphism | C | A | Sorbitol dehydrogenase |
| 2 | 4398569 | single nucleotide polymorphism | G | C | Intergenic region |
| 2 | 4432563 | insertion | C | CG | Hypothetical protein |
| 2 | 4516675 | single nucleotide polymorphism | T | C | 3-demethoxyubiquinol 3-hydroxylase |
| 2 | 5820881 | single nucleotide polymorphism | C | T | Inner membrane transport protein YnfM |
| 2 | 5862691 | single nucleotide polymorphism | A | G | glycosyltransferase family 1 protein |
| 2 | 5865580 | insertion | C | CG | O-antigen ligase WaaL |
| 3 | 70507 | deletion | AG | A | Intergenic region |
| 3 | 951537 | insertion | C | CG | Hypothetical protein |
| 3 | 958920 | single nucleotide polymorphism | C | T | Putative diguanylate cyclase DgcE |
| 3 | 1965635 | single nucleotide polymorphism | A | G | 5-oxoprolinase subunit C |
| 3 | 1966370 | deletion | AG | A | 5-oxoprolinase subunit A |
| 3 | 2188204 | single nucleotide polymorphism | A | G | Intergenic region |
| 3 | 2828940 | single nucleotide polymorphism | T | C | Translation initiation factor IF-3 |
| 3 | 3094356 | single nucleotide polymorphism | G | A | Transcription-repair-coupling factor |
| 3 | 3462822 | insertion | G | GC | Hypothetical protein |
| 3 | 3822391 | insertion | C | CT | Intergenic region |
| 3 | 4289904 | single nucleotide polymorphism | C | A | Sorbitol dehydrogenase |
| 3 | 4516675 | single nucleotide polymorphism | T | C | 3-demethoxyubiquinol 3-hydroxylase |
| 3 | 4716112 | single nucleotide polymorphism | C | G | preA |
| 4 | 70507 | deletion | AG | A | Intergenic region |
| 4 | 185577 | single nucleotide polymorphism | T | C | tldD |
| 4 | 356000 | complex | GCG | TT | Intergenic region |
| 4 | 450260 | single nucleotide polymorphism | T | C | Intergenic region |
| 4 | 493952 | deletion | TC | T | Intergenic region |
| 4 | 643225 | single nucleotide polymorphism | C | A | Intergenic region |
| 4 | 951537 | insertion | C | CG | nitrate reductase cytochrome subunit C |
| 4 | 958920 | single nucleotide polymorphism | C | T | Putative diguanylate cyclase DgcE |
| 4 | 1143181 | single nucleotide polymorphism | C | G | Intergenic region |
| 4 | 1143195 | single nucleotide polymorphism | A | T | Intergenic region |
| 4 | 1177651 | single nucleotide polymorphism | A | G | Intergenic region |
| 4 | 1180554 | single nucleotide polymorphism | G | T | nucleotidyl transferase containing domain |
| 4 | 1965539 | complex | AC | G | 5-oxoprolinase subunit C |
| 4 | 1965550 | insertion | C | CG | 5-oxoprolinase subunit C |
| 4 | 1965635 | single nucleotide polymorphism | A | G | 5-oxoprolinase subunit C |
| 4 | 2188204 | single nucleotide polymorphism | A | G | Intergenic region |
| 4 | 2707679 | single nucleotide polymorphism | C | T | Intergenic region |
| 4 | 2821258 | insertion | G | GC | N6 DNA methylase |
| 4 | 2828940 | single nucleotide polymorphism | T | C | Translation initiation factor IF-3 |
| 4 | 2994714 | single nucleotide polymorphism | C | A | Transcription-repair-coupling factor |
| 4 | 3094356 | single nucleotide polymorphism | G | A | cobK |
| 4 | 3258157 | deletion | GC | G | Hypothetical protein |
| 4 | 3312058 | single nucleotide polymorphism | C | G | Intergenic region |
| 4 | 3455609 | single nucleotide polymorphism | C | A | dhbf |
| 4 | 3462822 | insertion | G | GC | Hypothetical protein |
| 4 | 3549107 | insertion | G | GC | Intergenic region |
| 4 | 3549117 | insertion | G | GC | Intergenic region |
| 4 | 3822381 | multiple nucleotide polymorphism | GAC | ACG | Intergenic region |
| 4 | 3822391 | insertion | C | CT | Intergenic region |
| 4 | 4014283 | single nucleotide polymorphism | G | T | Intergenic region |
| 4 | 4029328 | single nucleotide polymorphism | C | A | Hypothetical protein |

*complex = combination of single nucleotide polymorphisms/multiple nucleotide polymorphisms
