# Supplementary material for: Transcriptomics-Driven Characterization of LUZ100, a T7-like Pseudomonas Phage with Temperate Features
Source: mSystems. 2023 Feb 16;8(2):e01189-22. doi: 10.1128/msystems.01189-22 (PMC10134795; doi:10.1128/msystems.01189-22)
Supplement: TABLE S4 [file msystems.01189-22-s0006.pdf]

**Supplementary Table S4**

| Gene | Position | strand | Amino acids | Putative function | T7 homologue | other BLASTP hit | % query coverage | E value | Percent Identity |
|---|---|---|---|---|---|---|---|---|---|
| *gp1* | 2-340 | + | 112 | hypothetical | - | - | - | - | - |
| *gp2* | 368-463 | + | 31 | hypothetical | - | - | - | - | - |
| *gp3* | 596-709 | + | 37 | hypothetical | - | - | - | - | - |
| *gp4* | 762-989 | + | 87 | hypothetical | - | QOI68779.1 | 54% | 4.00E-14 | 72.34% |
| *gp5* | 1009-1104 | + | 31 | hypothetical | - | - | - | - | - |
| *gp6* | 1117-1518 | + | 133 | hypothetical | - | WP_200593858.1 | 100% | 2.00E-81 | 88.72% |
| *gp7* | 1569-1667 | + | 32 | hypothetical | - | - | - | - | - |
| *gp8* | 1651-1935 | + | 94 | hypothetical | - | MCF6782036.1 | 100% | 1.00E-61 | 98.94% |
| *gp9* | 2147-2314 | + | 55 | hypothetical | - | - | - | - | - |
| *gp10* | 2321-2650 | + | 109 | transcriptional regulator, MarR family | - | WP_200593862.1 | 100% | 4.00E-70 | 97.30% |
| *tRNA* | 2658-2742 | - | - | tRNA-Leu-TAA | - | - | - | - | - |
| *gp11* | 2756-3829 | + | 357 | site-specific integrase | - | WP_100632527.1 | 100% | 0 | 99.16% |
| *gp12* | 3954-4067 | + | 37 | hypothetical | - | - | - | - | - |
| *gp13* | 4161-6686 | + | 841 | DNA-directed RNA polymerase (E.C.2.7.7.6) | T7 gp1 | - | 97% | 9.00E-173 | 38.55% |
| *gp14* | 6752-6964 | + | 70 | hypothetical | - | UHX60256.1 | 60% | 3.00E-04 | 52.27% |
| *gp15* | 7011-7667 | + | 218 | single-stranded DNA-binding protein | T7 gp2.5 | - | 52% | 1.00E-10 | 35.04% |
| *gp16* | 7667-8113 | + | 148 | endonuclease I | T7 gp3 | - | 88% | 8.00E-50 | 53.38% |
| *gp17* | 8110-8622 | + | 170 | N-acetylmuramoyl-L-alanine amidase | T7 gp3.5 | - | 86% | 3.00E-36 | 37.58% |
| *gp18* | 8659-8961 | + | 100 | putative DNA-binding protein | - | WP_200593770.1 | 80% | 7.00E-50 | 97.50% |
| *gp19* | 8972-9172 | + | 66 | hypothetical | - | WP_119522904.1 | 100% | 4.00E-36 | 98.48% |
| *gp20* | 9260-9916 | + | 218 | KilA-N domain-containing protein (DNA-binding) | - | WP_088515624.1 | 97% | 6.00E-91 | 59.83% |
| *gp21* | 9913-11568 | + | 551 | DNA primase/helicase | T7 gp4 | - | 92% | 8.00E-101 | 37.08% |
| *gp22* | 11608-13404 | + | 598 | DNA polymerase | T7 gp5 | - | 98% | 1.00E-159 | 42.51% |
| *gp23* | 13404-13616 | + | 70 | hypothetical | T7 gp5.5-5.7 | - | 90% | 1.00E-11 | 42.86% |
| *gp24* | 13616-13945 | + | 109 | hypothetical | - | WP_200593780.1 | 88% | 2.00E-11 | 98.97% |
| *gp25* | 13966-14676 | + | 236 | FAD-dependent thymidylate synthase | - | WP_212628436.1 | 100% | 4.00E-170 | 95.34% |
| *gp26* | 14669-14902 | + | 77 | hypothetical | - | WP_200593784.1 | 100% | 1.00E-46 | 100.00% |
| *gp27* | 14892-15674 | + | 260 | exonuclease | T7 gp6 | - | 98% | 3.00E-39 | 32.16% |
| *gp28* | 15662-15982 | + | 106 | hypothetical | T7 gp1.7 | - | 46% | 1.00E-08 | 39.62% |
| *gp29* | 15979-16281 | + | 100 | MazG-like family protein, nucleotide pyrophosphohydrolase protein | - | WP_100632557.1 | 100% | 9.00E-66 | 98.00% |
| *gp30* | 16271-16468 | + | 65 | Ribonucleoside-diphosphate reductase | - | WP_200593792.1 | 63% | 9.00E-16 | 90.24% |
| *gp31* | 16443-16640 | + | 65 | hypothetical | - | WP_212628430.1 | 100% | 3.00E-29 | 93.85% |
| *gp32* | 16706-17290 | + | 194 | hypothetical | - | MBX6273935.1 | 76% | 3.00E-102 | 95.97% |
| *gp33* | 17294-17578 | + | 94 | Putative virion tail assembly protein | T7 gp7.3 | - | - | - | - |
| *gp34* | 17591-19132 | + | 513 | head-to-tail connector protein | T7 gp8 | - | 95% | 1.00E-145 | 46.59% |
| *gp35* | 19125-19283 | + | 52 | hypothetical | - | WP_200593810.1 | 100% | 4.00E-25 | 94.34% |
| *gp36* | 19296-20072 | + | 258 | Capsid assembly protein | T7 gp9 | - | 64% | 3.00E-16 | 28.41% |
| *gp37* | 20081-20278 | + | 65 | hypothetical | - | WP_200593814.1 | 100% | 3.00E-24 | 100.00% |
| *gp38* | 20426-20617 | + | 63 | hypothetical | - | KXK67359.1 | 100% | 6.00E-38 | 98.41% |
| *gp39* | 20631-20765 | + | 44 | hypothetical | - | - | - | - | - |
| *tRNA* | 20831-20906 | + | - | tRNA-Asn-GTT | - | - | - | - | - |
| *gp40* | 21071-21199 | + | 42 | hypothetical | - | - | - | - | - |
| *gp41* | 21192-22145 | + | 317 | Major capsid protein | T7 gp10 | - | 95% | 8.00E-55 | 36.86% |
| *gp42* | 22166-22787 | + | 202 | Tail tubular protein A | T7 gp11 | - | 88% | 3.00E-25 | 30.77% |
| *gp43* | 22790-25186 | + | 798 | Tail tubular protein B | T7 gp12 | - | 99% | 4.00E-163 | 36.59% |
| *gp44* | 25147-25650 | + | 167 | Probable scaffold protein | T7 gp13 | - | 64% | 6.00E-05 | 29.20% |
| *gp45* | 25650-26240 | + | 196 | Putative internal virion protein | T7 gp14 | YP_009783945.1 | 51% (89%) | 0.13 (6e-17) | 28.3% (35.96%) |
| *gp46* | 26251-27963 | + | 570 | Putative internal virion protein | T7 gp15 | - | - | - | - |
| *gp47* | 27972-31604 | + | 121 | Putative internal virion protein | T7 gp16 | - | - | - | - |
| *gp48* | 31671-33623 | + | 650 | tail protein | T7 gp17 | - | 35% | 1.00E-16 | 29.44% |
| *gp49* | 33633-34043 | + | 136 | Putative tail-fibre assembly protein | - | WP_060738951.1 | 97% | 8.00E-08 | 37.23% |
| *gp50* | 34040-34267 | + | 75 | type II holin | T7 gp17.5 | QYW02342.1 | 36% (65%) | 0.01 (2e-07) | 33.33% (55.10%) |
| *gp51* | 34242-34475 | + | 77 | Terminase small subunit | T7 gp18 | QXV74670.1 | - (94%) | - (1e-09) | - (41.89%) |
| *gp52* | 34585-35010 | + | 141 | Putative restriction endonuclease | - | UHX60261.1 | 94% | 1.00E-13 | 36.76% |
| *gp53* | 35010-36704 | + | 564 | Terminase large subunit | T7 gp19 | - | 91% | 3.00E-164 | 49.53% |
| *gp54* | 36708-36845 | + | 45 | hypothetical | - | - | - | - | - |
| *gp55* | 36908-37030 | + | 40 | hypothetical | - | - | - | - | - |
| *gp56* | 37075-37272 | + | 65 | hypothetical | - | - | - | - | - |
