# Supplementary material for: Transcriptomics-Driven Characterization of LUZ100, a T7-like Pseudomonas Phage with Temperate Features
Source: mSystems. 2023 Feb 16;8(2):e01189-22. doi: 10.1128/msystems.01189-22 (PMC10134795; doi:10.1128/msystems.01189-22)
Supplement: TABLE S5 [file msystems.01189-22-s0007.pdf]

Supplementary Table S5

Terminator	Position	Strand	Sequence
T1	673-772	+	GCCCGTAGGTAACACAAACGGCAAGGATTGCCGGTAATCCCCAAGGATGGTGGATGCCT**C**AAGGCACATTGACTGGGTCATTCCCAGGATCAGTGCGCCT
T2	1510-1609	+	GCTGCCTGATTGATCATAAGTAGTGGCCTTCTAATGCCATTTCGATATTAGAGGGCCAC**T**TGAAAGAAAGGCTTCTAGCCCCCAACCATTTGCGCCCGGA
T3	2504-2603	+	TCCCGAGTTCACCGGCTGAACAAGCCAGGGATGGATCTGGTTGTGGCTGCAGAAGACCC**G**ATGGAACGCCGCCGTAAGGTAGTCAAGCTGACCCCGAAAG
T4	3926-4025	+	GTCTTGCATCCGACTATCCACTGGCACATTGAATAACCCGGCATCGTCCGGGTTTTCTT**T**TCCCATCAACTATCCACTGTCGCAACCTTTGCAGACCGCT
T5	7652-7751	+	CGACGAGGACTTCTGATGGCTGCTCCCCGAGAGGCCGGGCTTCGGCTCGGCTTCCGGTC**G**GGACTTGAGGAGAAGATCGCCCGAGAACTGGATGCCCACG
T6	8624-8723	+	CAATTACCACACAGGTATGGAAGACCGCCCCCGGTATGCCCAGCGCTGCCGGGGGTTTC**T**GTTTCAAGACCCACGACAACTGTGAGGAGAACCCCATGAC
T7	9182-9281	+	ACGATTCACCGGGTCAGCTACCAACGAACACCCATAGCCCGCCTCTGAGCGGGCTTCGT**C**TTTTCTGGAGGACAGAACATGAGCAAGCAACTGACTGTAT
T8	9984-10083	+	GACAAAGGTTGGGGGAAGTGCTTCTCGTGCGGGAAGAACTTCAACGAGGAGGCGTCGGA**C**TCAACACCCAGAGGTAAACAGCAAGTGGCAGCAGTGAAAG
T9	10619-10718	+	AAGTGATCCAAGCAATCTGGAACGCAAAGGAGTACCGGCCCGATGGGCTGGTCTCCATC**G**ACGACATCCTCGACGAGGTCGAGAAGCCCATCGAATGGGG
T10	13028-13127	+	AGCGGCTACGCGAAGACGTTGCGTCAGCCGTGAAAAAGCGGGGCTACCTCGTCGGGATC**G**ACGGGCGACACCTGCACATCCGATCCGACCACGCCGCCCT
T11	17137-17236	+	GTCCGAGGTGTTCGAGGTGATGAAGACCTTCGCGCTGCTCCACGGGGCAAGGGGTTGTG**A**GTTCGGCACACGCGCAGCCAGCAACAAAGCGGCCATCCGA
T12	19140-19239	+	CAACCGAAAGTCACCAGCCAGGAAACCAAAGGGGGTCGCCGCAAGACGGCCCCTTCAGT**T**TCTGCGGCTGAAACCAACAAGCAACCCCAAGAGGCCAAGC
T13	20323-20422	+	CTGGTGCTCACCCTCCTGCGTGACGACAAGTTGCGCCAGGAAGGTGAGCGCAAGGTGCG**C**CTGGAGGCGATGGAGGCCGAGCAACAGCGCCGTGGAGAAG
T14	20769-20868	+	CACGCTCAAATCGTGGGGCCGCTAGGCAGCTTGCTCCGTCTGCCGAATCACGGAGCTTT**C**ACTGGCATGTAGCTCAGATGGTAGAGCACCCGGCTGTTAA
T15	20942-21041	+	TAGCACGTCGGGAGACGTTACTGGGCAACCCCTACCTCGAACGACGAGTGCCTCAAGTC**G**CACTGGCAGCAAAGCCGGTGACTTAGCACCCAAAGCATTT
T16	22135-22234	+	CTGTTGCGTAAGCAACTATCCACCATCGCATAGGAGGGCCAGAAATGGCTCTCCTATTT**T**TTTCTTATGGGGGTTCCATGATTTCTCCAACCACTGAGCT
T17	25333-25432	+	TTCGTGGCAGTCGATGCGCAGGACAAGCCTCAGATCATCTGGGGCACCTGCCCATCCGA**T**GAACCCTACCTGGGGTACGTCTGGATGATGGCCACCGACG
T18	28067-28166	+	CGAAGCGCCCCAGGAAGCTGCCCCGGCCCCGACTGATTACCAGTATGGGGCTGTGGCCG**A**GGACATCGACCCCGATACCCTGGCCAGCAACCAAGACTGG
T19	31593-31692	+	TGGGATCAGTAACAACCATTCGTTCAACGCACAGGGGCGGCCTTCGGGTCGCCCTTTTT**C**TTTGTCTGGAGGAAAAGCATGGCTCTTGCACGAGTCACCT
T20	36691-36790	+	GGATGGTTATGTAGAACATGGATGTGACTGAGGGGAGCAGGAGGCTCCTCTCGGTCTCT**T**TGCAAGATCGCTGCAAGATCGACGACGTTCAATTACCACA
T21	37057-37156	+	CCTTGGTTTTTGCCAAAAATGCGAGACGGTATAGCGCCAACCCTGGCCCTGGAGTTCCC**C**CCGGTGGGGTGGGCACCTGGTCTGCGAATCGAGGCCGGTG
T22	37133-37232	+	TGGTCTGCGAATCGAGGCCGGTGCCGGGGGCTGCCGGTGCTGGTCGGGGGGCTGGCGAC**A**CAAGCCACGACACAAGATGCCCGCCAAGCCGCGCCGTTGC
