# Supplementary material for: Transcriptomics-Driven Characterization of LUZ100, a T7-like Pseudomonas Phage with Temperate Features
Source: mSystems. 2023 Feb 16;8(2):e01189-22. doi: 10.1128/msystems.01189-22 (PMC10134795; doi:10.1128/msystems.01189-22)
Supplement: TABLE S6 [file msystems.01189-22-s0008.pdf]

Supplementary Table S6

Phage	NCBI Accession Number	RNAP specificity loop	RNAP recognition loop
LUZ100		KMLTERIDLTFGGSRHLLTV---AVSPTTEL	FIESCESGKAGRKHSA
PPpW-4	NC_023005.1	VQSGRRIDMVILGDVRLQITVKSS-SKDDKI	WTAH-QMNSVRRKSVA
Phi15	NC_015208.1	VQQAKRIDMILMGDVRLTATVLHQ---QDQI	WVEH-QMKNVRRKSVA
PFP1	NC_047997.1	VNDKVRVDLIFMGEVRLQTTVMHDDRPQNKI	WVEH-QMTKVRRKSVA
71PfluR64PP	MH179475.2	VNDKVRVDLIFMGEVRLQTTVMHDDRPQNKI	WVEH-QMTKVRRKSVA
22PfluR64PP	NC_047965.1	VNDKVRVDLIFMGEVRLQTTVMHDDRPQNKI	WVEH-QMTKVRRKSVA
67PfluR64PP	MH179478.2	VNDKVRVDLIFMGEVRLQTTVMHDDRPQNKI	WVEH-QMTKVRRKSVA
UNO-SLW1	NC_047873.1	VADKVRIDLIFMGDVRLQTTVLQGDKKENKI	WKHH-QMTKVRRKSVA
UNO-SLW4	KX449363.1	VADKVRIDLIFMGDVRLQTTVLQGDKKENKI	WKHH-QMTKVRRKSVA
UNO-SLW3	KX449362.1	VADKVRIDLIFMGDVRLQTTVLQGDKKENKI	WKHH-QMTKVRRKSVA
UNO-SLW2	KX449361.1	VADKVRIDLIFMGDVRLQTTVLQGDKKENKI	WKHH-QMTKVRRKSVA
philBB-PF7A	NC_015264.1	VADKVRVDLIFMGDVRLQTTVLQTDRGDNKI	WTEH-QMTKVRRKSVA
BIM-BV-46	MT094431.1	VADKVRIDLIFLGDVRLQTSVMQGDKPTNKI	WKHH-QMTKVRRKSVA
Pf-10	NC_027292.1	VADKVRIDLIFLGDVRLQTSVMQGDKPTNKI	WKHH-QMTKVRRKSVA
Phi-S1	NC_021062.1	VADKVRIDLIFLGDVRLQTSVMQGDKPTNKI	WKHH-QMTKVRRKSVA
PPPL-1	NC_028661.1	IPATRRVDLMFLGDIRLQATVNLRDDGTNKI	WIEH-QTTKVRRKHVA
shl2	NC_048200.1	IPATRRVDLMFLGDIRLQATVNLRDDGTNKI	WIEH-QVTKVRRKHVA
Henninger	MG775258.1	IPETRRIDLMFLGDIRIQSTVTVRDS--DKI	WIEH-QTTKVRRKHVA
pf1-ERZ-2017	MG250485.1	IPETRRIDLMFLGDVRIQATVTVRDS--DKI	WIEY-QTTKVRRKHVA
PhiPsa17	KR091952.1	IPETRRIDLMFLGDVRIQATVTVRDS--DKI	WIEH-QTTKVRRKHVA
KNP	KY798121.1	IPETRRIDLMFLGDVRIQATVTVRDS--DKI	WIEH-QTTKVRRKHVA
gh-1	NC_004665.1	IPETRRIDLMFLGDVRIQATVTVRDS--DKI	WIEH-QTTKVRRKHVA
phiPSA2	KJ507099.1	IPETRRIDLMFLGDVRIQATVTVRDS--DKI	WIEH-QTTKVRRKHVA
WRT	KY798120.1	IPETRRIDLMFLGDVRIQATVTVRDS--DKI	WIEH-QTTKVRRKHVA
T7	NC_001604.1	KPIQTRLNLMFLGQFRLQPTINTNK--DSEI	WFEE-VKAKRGKRPTA
