# Supplementary material for: Transcriptomics-Driven Characterization of LUZ100, a T7-like Pseudomonas Phage with Temperate Features
Source: mSystems. 2023 Feb 16;8(2):e01189-22. doi: 10.1128/msystems.01189-22 (PMC10134795; doi:10.1128/msystems.01189-22)
Supplement: TABLE S7 [file msystems.01189-22-s0009.pdf]

**Supplementary Table S7**

| TSS location | TTS | TTS location | gene content |
|---|---|---|---|
| 405 | T1 | 732 | gp03 |
| 606 | T1 | 732 | gp03 |
| 2273 | T3 | 2563 | - |
| 2273 | T4 | 3985 | gp10 \| gp11 |
| 4100 | T5 | 7711 | gp13 \| gp14 \| gp15 |
| 4100 | T6 | 8683 | gp13 \| gp14 \| gp15 \| gp16 \| gp17 |
| 6756 | T5 | 7711 | gp14 \| gp15 |
| 6756 | T6 | 8683 | gp14 \| gp15 \| gp16 \| gp17 |
| 6756 | T7 | 9241 | gp14 \| gp15 \| gp16 \| gp17 \| gp18 \| gp19 |
| 6756 | T8 | 10043 | gp14 \| gp15 \| gp16 \| gp17 \| gp18 \| gp19 \| gp20 |
| 6756 | T9 | 10678 | gp14 \| gp15 \| gp16 \| gp17 \| gp18 \| gp19 \| gp20 |
| 6801 | T5 | 7711 | gp15 |
| 6801 | T6 | 8683 | gp15 \| gp16 \| gp17 |
| 6801 | T7 | 9241 | gp15 \| gp16 \| gp17 \| gp18 \| gp19 |
| 6801 | T8 | 10043 | gp15 \| gp16 \| gp17 \| gp18 \| gp19 \| gp20 |
| 6801 | T9 | 10678 | gp15 \| gp16 \| gp17 \| gp18 \| gp19 \| gp20 |
| 6801 | T10 | 13087 | gp15 \| gp16 \| gp17 \| gp18 \| gp19 \| gp20 \| gp21 |
| 8644 | T7 | 9241 | gp18 \| gp19 |
| 8644 | T8 | 10043 | gp18 \| gp19 \| gp20 |
| 8644 | T9 | 10678 | gp18 \| gp19 \| gp20 |
| 8644 | T10 | 13087 | gp18 \| gp19 \| gp20 \| gp21 |
| 8786 | T7 | 9241 | gp19 |
| 8786 | T8 | 10043 | gp19 \| gp20 |
| 8786 | T9 | 10678 | gp19 \| gp20 |
| 8786 | T10 | 13087 | gp19 \| gp20 \| gp21 |
| 16410 | T11 | 17196 | gp31 |
| 16410 | T12 | 19199 | gp31 \| gp32 \| gp33 \| gp34 |
| 16410 | T13 | 20382 | gp31 \| gp32 \| gp33 \| gp34 \| gp35 \| gp36 \| gp37 |
| 16410 | T14 | 20828 | gp31 \| gp32 \| gp33 \| gp34 \| gp35 \| gp36 \| gp37 \| gp38 \| gp39 |
| 16410 | T15 | 21001 | gp31 \| gp32 \| gp33 \| gp34 \| gp35 \| gp36 \| gp37 \| gp38 \| gp39 \| tRNA-Asn |
| 16410 | T16 | 22194 | gp31 \| gp32 \| gp33 \| gp34 \| gp35 \| gp36 \| gp37 \| gp38 \| gp39 \| tRNA-Asn \| gp40 \| gp41 |
| 20635 | T14 | 20828 | gp39 |
| 20635 | T15 | 21001 | gp39 \| tRNA-Asn |
| 20635 | T16 | 22194 | gp39 \| tRNA-Asn \| gp40 \| gp41 |
| 36799 | T21 | 37116 | gp55 |
| 36799 | T22 | 37192 | gp55 |
| 36799 | T1 | 732 | gp55 \| gp56 \| gp01 \| gp02 \| gp03 |
