# Supplementary material for: Transcriptomics-Driven Characterization of LUZ100, a T7-like Pseudomonas Phage with Temperate Features
Source: mSystems. 2023 Feb 16;8(2):e01189-22. doi: 10.1128/msystems.01189-22 (PMC10134795; doi:10.1128/msystems.01189-22)
Supplement: TABLE S1 [file msystems.01189-22-s0003.pdf]

Supplementary Table S1

PRIMER LIST

Nr.	Primer name	sequence (5'-3')	Use
1	PaLo41_GyrB_F	ccgtggtggtagacctgttc	gDNA contamination test
2	PaLo41_GyrB_R	gcaagttcgacgacaacacc	gDNA contamination test
3	LUZ100_TerL_F	ccgtgaagtccgtgggtatc	gDNA contamination test
4	LUZ100_TerL_R	gtcgaccaagtagagccacc	gDNA contamination test
5	LUZ100_P3_F	tctatgctagactggattgtcgaaccgaatgatgttttactgtgggtgtgga	fluorescence assays (promoter)
6	LUZ100_P3_R	ctgctccacacccacagtaaaacatcattcggttcgacaatccagtctagca	fluorescence assays (promoter)

INSERT LIST

Nr.	insert name	sequence (5'-3')	use
1	LUZ100_P3	atgctagactggattgtcgaaccgaatgatgttttactgtgggtgtggag	fluorescence assays
2	Pem7	ttgttgacaattaatcatcggcatagtatatcggcatagtataatacgacaaggtgaggaactaaacc	fluorescence assays
3	BCD2v	gcccaagttcacttaaaaaggagatcaacaatgaaagcaattttcgtactgaaacatcttaatcatgctaaggaggt	fluorescence assays
4	msfGFP	atgatcatgggaattcataaaggtgaagaactgttcaccggtgttgttccgatcctggttgaactggatggtgatgttaacggccacaaattctctgttcgtggtgaaggtgaaggtgatgcaaccaacggtaaactgaccctgaaattcatctgcactaccggtaaactgccggttccatggccgactctggtgactaccctgacctatggtgttcagtgtttttctcgttacccggatcacatgaagcagcatgatttcttcaaatctgcaatgccggaaggttatgtacaggagcgcaccatttctttcaaagacgatggcacctacaaaacccgtgcagaggttaaatttgaaggtgatactctggtgaaccgtattgaactgaaaggcattgatttcaaagaggacggcaacatcctgggccacaaactggaatataacttcaactcccataacgtttacatcaccgcagacaaacagaagaacggtatcaaagctaacttcaaaattcgccataacgttgaagacggtagcgtacagctggcggaccactaccagcagaacactccgatcggtgatggtccggttctgctgccggataaccactacctgtccacccagtctaaactgtccaaagacccgaacgaaaagcgcgaccacatggtgctgctggagttcgttactgcagcaggtatcacgcacggcatggatgaactctacaaataa	fluorescence assays

VECTOR LIST

Nr.	Vector	Insert	use
1	pBGDes	BCD2-msfGFP	fluorescence assays (promoter), negative control
2	pBGDes	LUZ100_P3-BCD2-msfGFP	fluorescence assays (promoter)
3	pBGDes	Pem7-BCD2-msfGFP	fluorescence assays (promoter), positive control
