# Supplementary material for: Transcriptomics-Driven Characterization of LUZ100, a T7-like Pseudomonas Phage with Temperate Features
Source: mSystems. 2023 Feb 16;8(2):e01189-22. doi: 10.1128/msystems.01189-22 (PMC10134795; doi:10.1128/msystems.01189-22)
Supplement: TABLE S2 [file msystems.01189-22-s0004.pdf]

Supplementary Table S2

A.

RAW READ METRICS				
**Sample**	**Total number of reads**	**Mean read quality score**	**Mean read length (bp)**	**Read length N50 (bp)**
tϕ, enriched	1,611,144	10.6	690.7	652
tϕ, control	1,265,750	10.9	1,558.0	2,847
t0, enriched	2,578,503	9.9	640.3	606
t0, control	1,604,147	10.1	1,022.7	1,718

B.

PROCESSED READ METRICS					
**Sample**	**Total number of reads**	**Mean read quality score**	**Mean read length (bp)**	**Read length N50 (bp)**	**Longest read (bp)**
tϕ, enriched	1,471,568	11.5	500.7	579	7,011
tϕ, control	1,136,712	11.6	1,381.0	2,776	7,563
t0, enriched	2,156,469	10.8	473.2	588	3,439
t0, control	1,365,984	11.1	863.5	1,939	5,573

C.

MAPPING METRICS (after clipping)						
**Sample**	**Total number of reads**	**Number of mapped reads**	**Number of reads mapped to phage**	**Number of reads mapped to host**	**Number of reads mapped to in vitro transcribed spike-in**	**Average percent identity**
tϕ, enriched	1,266,992	1,194,222	144,891	1,044,020	5,311	92.40
tϕ, control	1,022,372	930,712	26,035	904,341	336	92.50
t0, enriched	1,778,068	1,604,100	31	1,601,257	2,812	91.80
t0, control	1,218,963	885,378	9	885,036	333	92.80
